# Recombinant serralysin metalloproteases D enhances the intracellular replication of infectious bovine rhinotracheitis virus

**DOI:** 10.3389/fmicb.2025.1567288

**Published:** 2025-05-09

**Authors:** Longfei Yan, Yanran Li, Jiancheng Qi, Li Ren, Xueke Zhou, Liping Gou, Zhicai Zuo

**Affiliations:** ^1^Key Laboratory of Animal Disease and Human Health of Sichuan Province, College of Veterinary Medicine, Sichuan Agricultural University, Chengdu, Sichuan, China; ^2^State Key Laboratory of Virology, Taikang Center for Life and Medical Sciences, College of Life Sciences, Wuhan University, Wuhan, China

**Keywords:** infectious bovine rhinotracheitis virus, *Serratia marcescens* protease, co-infection, virus replication, bacteria-virus interplay

## Abstract

Infectious bovine rhinotracheitis virus (IBRV) and *Serratia marcescens* co-infection are commonly observed in the respiratory tract of cattle subjected to respiratory diseases. However, the potential effects of proteases from *Serratia marcescens* on the IBRV infection remain poorly understood. In this study, we investigated the role of recombinant serralysin-like protease D (rSPD) in modulating IBRV infection in Madin-Darby bovine kidney (MDBK) cells. Our findings demonstrate that rSPD enhances IBRV replication and exacerbates the cytopathic effects of the virus on MDBK cells. Quantification of IBRV *gB* gene copy numbers using fluorescence quantification PCR (FQ-PCR) revealed that rSPD promotes viral replication during the intracellular stage, without affecting viral adsorption, entry, or directly interacting with viral particles. The transcriptomic analysis further demonstrated that rSPD suppresses innate immune responses while amplifying inflammatory pathways in IBRV-infected MDBK cells. Gene Ontology (GO) and KEGG enrichment analysis identified significant enrichment of differentially expressed genes (DEGs) in key signaling pathways, including JAK–STAT, NOD-like receptor, Toll-like receptor, TNF, NF-κB, and MAPK pathways. Notably, rSPD downregulated genes associated with innate immunity, such as *ISG15*, *OAS2*, *IFIT1*, *IFIT2*, *IFIT3*, *MX1*, *RSAD2*, *MX2*, *SAA3*, *DDX58*, *IFI44*, and *IRF1*, suggesting that rSPD suppresses host antiviral defenses. Conversely, rSPD upregulated genes involved in inflammatory response, including *IL-6*, *IL-8*, *CCL2*, *CX3CL1*, *CCL3*, and *CXCL3*, indicating that rSPD may exacerbate cellular damage and promote viral replication by inducing excessive inflammatory responses. These findings provide novel insights into the interplay between bacterial proteases and viral infections, highlighting the potential role of bacterial proteases in exacerbating viral pathogenesis and offering a foundation for further research into therapeutic strategies targeting bacterial-viral interactions.

## Introduction

Infectious bovine rhinotracheitis virus (IBRV), also referred to as bovine herpesvirus-1 (BoHV-1), is a major pathogen in cattle, responsible for significant economic losses worldwide derived from its association with respiratory and reproductive diseases, pregnancy wastage, and the costs of implementing control measures (Engdawork and Aklilu, 2024; Madin et al., 1956). As a member of the *Herpesviridae* family, IBRV is characterized by a large, double-stranded, linear DNA genome and a relatively short reproductive cycle (Muylkens et al., 2007). The viral life cycle consists of several key stages, including host cell adsorption, invasion, intracellular replication, and release. Notably, the severity of clinical diseases caused by IBRV is closely linked to its replication efficiency within host cells (Yatim and Albert, 2011). A range of factors has been identified that modulate the replication of IBRV by influencing various stages of its life cycle. For example, studies have demonstrated that low concentrations of ivermectin (6–25 nM) can effectively inhibit IBRV replication and reduce viral titers by targeting specific viral or host factors (Wang et al., 2023). Given the significant role of replication in IBRV pathogenesis, a deeper understanding of the mechanisms regulating its replication could provide valuable insights for disease prevention and treatment.

Recent studies have demonstrated that the presence of co-infecting pathogens can significantly influence the proliferation of viruses. For example, co-infection with viruses such as bovine coronavirus and bovine respiratory syncytial virus was also found to enhance viral load and replication by modulating the host immune response (Makoschey and Berge, 2021). Similarly, bacterial infections can exacerbate viral replication and host damage, often through the secretion of proteases. For instance, proteases secreted by respiratory-colonizing bacteria, such as *Staphylococcus aureus*, have been found to enhance the infectivity and pathogenicity of influenza virus in mice models (Mikušová et al., 2022). Likewise, Ami et al. reported that low-pathogenicity bacteria exacerbated SARS-CoV infection in a BALB/c mouse model by inducing a mild inflammatory response through elastase secretion (Ami et al., 2008). In addition to proteases, bacterial effector proteins, such as NleE from enteropathogenic *Escherichia coli* (EPEC), have been identified as key modulators of viral replication. NleE inhibits the activation of TBK1, thereby suppressing the innate antiviral response of the host and promoting viral proliferation (Rosadini and Kagan, 2015). Despite these findings, research on the impact of bacterial co-infections on IBRV replication in the bovine respiratory tract remains limited. Existing studies primarily focus on viral pathogenesis or bacterial effects in isolation, with limited investigation into how bacterial components influence viral replication at the molecular level during co-infection. This gap in knowledge is significant, as co-infections frequently result in more severe disease outcomes than infections caused by either pathogen alone (Asner et al., 2014). However, the cellular and molecular interplay between viral and bacterial factors remains poorly understood and inadequately characterized.

*Serratia marcescens* is a gram-negative bacterium commonly found in diverse environments, including soil, water, plants, animals, and insects (Cosimato et al., 2024). This bacterium secretes multiple extracellular enzymes that act as virulence factors, such as nucleases, chitinases, proteases, and lipase, which contribute to tissue damage and modulate host immune responses. Among these virulence factors, serralysin metalloproteases D (SPD) is one of the most abundant extracellular enzymes. SPD is secreted via the type I secretion system (T1SS), which utilizes the LipB-LipC-LipD transporters (Petersen and Tisa, 2013). SPD plays a critical role in the pathogenic mechanisms of *Serratia marcescens* by inducing inflammatory responses, disrupting cellular structures, and degrading host immune components to evade immune defenses (Carroll and Maharshak, 2013; Miyoshi and Shinoda, 2000). Research has demonstrated that SPD enhances bacterial survival and proliferation by degrading immunoglobulins and complement proteins, thereby impairing the host immune system’s ability to recognize and respond effectively to *S. marcescens* infections (Maeda and Molla, 1989). For instance, purified *Serratia* protease PrtA has been shown to damage over 50% of fibroblasts within 1 h of incubation (Petersen and Tisa, 2014). Conversely, mutant strains lacking this 56 kDa metalloprotease exhibited significantly reduced cytotoxicity toward HeLa cells (Marty et al., 2002). Beyond its role in bacterial pathogenies, SPD has also been found to enhance the proliferation and lethality of viruses. For example, Akaike et al. (1989) reported that a 56 kDa protease from *Serratia marcescens* enhanced influenza virus replication and increased its lethality in mice. Similarly, *in vivo* studies revealed that *Serratia marcescens* proteases significantly elevated influenza virus titers and exacerbated infection symptoms in mice models (Dubin et al., 2013). These findings suggest that SPD may influence viral replication and pathogenesis. However, whether SPD affects the replication capacity of IBRV remains unclear.

Traditionally, *Serratia marcescens* has been regarded as a pathogen associated with mastitis in dairy cows (Liang et al., 2023). In recent years, our laboratory has frequently isolated *Serratia marcescens* from the oral or nasal cavities of beef cattle suffering from respiratory diseases. Based on these findings, we hypothesized that SPD may influence the replication of IBRV. To test this hypothesis, we investigated whether recombinant SPD (rSPD) affects the proliferation of IBRV in Madin-Darby bovine kidney (MDBK) cells. Additionally, we conducted a preliminary exploration of the potential molecular mechanisms underlying this interaction using transcriptomic analysis. Our study is the first to report the effects of SPD on the replication of IBRV, which may contribute to the development of new strategies for mitigating the impact of co-infections involving *S. marcescens* and IBRV in bovine populations.

## Materials and methods

### Cells and virus strain

The MDBK cell line was obtained from the China General Microbiological Culture Collection Center (CGMCC). MDBK cells were maintained in Dulbecco’s Modified Eagle Medium (DMEM; Solarbio, Beijing, China) supplemented with 10% fetal bovine serum (FBS; TransGen, Beijing, China) and 1% penicillin–streptomycin (Solarbio, Beijing, China). Cells were cultured at 37°C in a humidified incubator with 5% CO_2_. IBRV was clinically isolated and stored in our laboratory. The virus was propagated in MDBK cells and subsequently used for viral titer determination (De Martino et al., 2003).

### Bacterial strain and rSPD preparation

The *Serratia marcescens* strain 6 M-6 was isolated from oral swabs of diseased cattle exhibiting respiratory symptoms on a regional beef cattle farm in Sichuan Province, China. The recombinant protein expression bacteria BL21-6 M6 was engineered and stored in our laboratory.

To prepare rSPD, the BL21-6 M6 strain was cultured overnight, and bacterial cells were harvested by centrifugation and subsequently lysed. The inclusion bodies were denatured using urea, and metal ions were removed through gradient dialysis. The target protein was purified using a nickel affinity column, followed by gradient refolding at low temperatures to restore its functional structure. The activity of the purified proteins was verified using the forinol method. Protein concentrations were then measured, and the purified protease was flash-frozen in liquid nitrogen before being stored at −80°C for further use.

### Cell viability assay

The CCK-8 assay kit (Beyotime, Shanghai, China) was employed to assess the viability of MDBK cells according to its manufacturer. Briefly, cells in the logarithmic growth phase were harvested to prepare a cell suspension at a density of 7 × 10^3^ cells/mL. A total of 100 μL of the cell suspension was seeded into each well of a 96-well plate and incubated for 24 h, allowing the cells to reach over 90% confluency. After incubation, the medium in each well was discarded, and rSPD was added at final concentrations of 0, 12.5, 25, 50, 100, and 200 μg/mL. Each concentration was tested in 5 replicates, with 1 blank well serving as a control. The cells were incubated for another 24 h. Subsequently, 110 μL of cell maintenance solution mixed with CCK-8 reagent (10:1) was added to each well. The plate was then protected from light and incubated at 37°C in 5% CO_2_ for 45 min. The optical density (OD) at 450 nm of each well was measured using a microplate reader. Cell viability (%) was calculated using the formula: Viability (%) = [(OD_sample_ − OD_blank_)/(OD_control_ − OD_blank_)] × 100.

### Viral titer assay

MDBK cells were cultured in 96-well plates and infected with serial 10-fold dilutions of IBRV. After a 1 h incubation at 37°C, the medium was replaced with fresh DMEM supplemented with 2% FBS. Viral titers were measured 48 h post-inoculation using endpoint dilution analysis. The 50% tissue culture infectious dose (TCID₅₀) was calculated using the Reed-Muench method (Thakur and Fezio, 1981).

### Plaque formation assay

MDBK cells were seeded into a 12-well plate at an initial density of 1 × 10^5^ cells per well and cultured for 24 h. The cells were then infected with 1 mL of IBRV (100 TCID₅₀/mL) per well. After a 1 h incubation at 37°C, the inoculum was replaced with rSPD diluted in a maintenance medium (DMEM supplemented with 1.5% agarose). Following a 24 h incubation, the solidified agarose was carefully removed. The cells were fixed with 10% formalin and stained with 1% crystal violet. Plaque-forming units (PFUs) in each well were subsequently quantified.

### Immunofluorescence assay

MDBK cells were cultured in 24-well plates containing pre-loaded coverslips for 24 h. Each well was then infected with 500 μL of IBRV (100 TCID₅₀/mL) and incubated for 1 h at 37°C. After the inoculum was removed, a maintenance medium containing varying concentrations of rSPD was added. The cell coverslips were fixed with 4% formaldehyde (Beyotime, Shanghai, China) for 10 min at −20°C and stained with IBRV polyclonal antiserum (VMRD, Washington, United States) for 1 h at 37°C. The slides were then gently rinsed with PBS buffer using a was bottle. The coverslips were then mounted in an anti-fluorescence quencher containing DAPI (Beyotime, Shanghai, China), observed, and captured under a fluorescence microscope (OLYMPUS BX53, Japan).

### Viral replication assay

MDBK cells were cultured in 6-well plates and infected with IBRV at a multiplicity of infection (MOI) of 0.1. After a 2 h incubation at 37°C, the cells were washed 3 times with PBS. DMEM supplemented with 2% FBS and containing rSPD (200 μg/mL) or PBS was then added to the wells. The cells were incubated at 37°C for 24 h. The supernatant and DNA from each group were collected. The copy number of the IBRV *gB* gene was quantified using a quantitative real-time PCR Kit (TransGen, Beijing, China), and CFX96 Touch Real-Time PCR Detection System (Bio-Rad, CA, United States). Additionally, the change in viral titers in the supernatant was determined using the Reed-Muench method.

### Virus binding assay

MDBK cells were seeded into 6-well plates and incubated with DMEM containing IBRV (0.1 MOI) at 4°C for 2 h in the presence of rSPD (200 μg/mL). The temperature condition was maintained to allow the virus to bind to the cells without internalization. After incubation, the cells were washed 3 times with pre-chilled PBS to remove unbound particles. The cells were then cultured in DMEM at 37°C for 24 h. The copy number of IBRV *gB* gene in the samples were quantified using qPCR, and viral titers were determined using the Reed-Muench method.

### Virus internalization assay

MDBK cells were seeded into 6-well plates and incubated with DMEM containing IBRV (0.1 MOI) at 4°C for 2 h. Following incubation, the cells were washed 3 times with PBS to remove unbound virus particles. Subsequently, rSPD (200 μg/mL) was added, and the cells were incubated at 37°C for 2 h to facilitate virus internalization. Afterward, the cells were washed with PBS to remove any remaining extracellular virus or compounds and were further cultured in DMEM at 37°C for 24 h. The copy number of IBRV *gB* gene were quantified by qPCR and viral titers were determined using the Reed-Muench method.

### Direct effect test of rSPD and IBRV

IBRV (0.1 MOI) was mixed with rSPD in DMEM, and the mixture was incubated at 37°C for 2 h. After incubation, the mixture was added to MDBK cells and further incubated at 37°C with 5% CO_2_ for 2 h. The supernatant was then discarded, and the cells were washed 3 times with PBS to remove any residual viruses or compounds. A cell maintenance solution was subsequently added, and the cells were cultured at 37°C for 24 h. The copy number of IBRV gB gene were quantified by q-PCR and viral titers were determined using the Reed-Muench method.

### Transcriptome sequencing and transcriptomic analysis

The transcriptome sequencing was performed by Novogene Co., Ltd. (Beijing, China). Briefly, total RNA was extracted from MDBK cells in each sample using the Trizol reagent (TransGen, Beijing, China), following the manufacturer’s protocol. The concentration and integrity of the extracted RNA were evaluated using a NanoDrop ND-2000 spectrophotometer and an Agilent Bioanalyzer 2,100 (Agilent Technologies, Santa Clara, United States). Only samples with an RNA integrity number (RIN) greater than 0.8 were selected for sequencing. They, complementary DNA libraries were prepared using the Illumina TruSeq RNA Sample Prep Kit, yielding an average fragment size of 150 bp (excluding adaptors). Library quality was assessed using the Agilent 2,100 Bioanalyzer and ABI StepOne Plus Real-Time PCR System (Thermo Fisher; Waltham, MA, United States). All libraries demonstrated a narrow size distribution with a peak around 275 bp and an effective concentration of at least 1.5 nM. Then, the libraries were added to the Illumina Casava 1.8 platform and the resulting RNA-Seq FASTQ files were aligned to the bovine genome using Hisat2 (Kim et al., 2019), with reference genome data obtained from the Ensembl Bovine Genome Database.^1^ The resulting binary alignment/map (BAM) files were processed using Cufflinks (Trapnell et al., 2012) to quantify transcript abundance and identify mRNA isoforms. StringTie (v1.3.3b) was employed to assemble the mapped reads using a reference-based strategy (Pertea et al., 2015). Transcript expression levels were quantified in terms of count, and Principal Component Analysis (PCA) was performed based on these count values. The expression levels of transcripts were analyzed using the Deseq2 package (Love et al., 2014) based on their counts. Gene Ontology (GO) and Kyoto Encyclopedia of Genes and Genomes (KEGG) enrichment analyses of the differentially expressed genes (DEGs) were performed using the ClusterProfiler package (Wu et al., 2021). The PPI network was constructed using data from the STRING database,^2^ and the network was visualized with Cytoscape.^3^

### Quantitative reverse transcription PCR analysis

qRT-PCR was employed to quantify the expression levels of genes in MDBK cells. Briefly, total RNA was extracted from MDBK cells in each sample using the Total RNA Rapid Extraction Kit (Tiangen, Beijing, China) and quantified using a NanoDrop (Thermo Fisher, Waltham, United States). The extracted RNA was reverse-transcribed into cDNA using HiScript III RT Super Mix (Vazyme, Nanjing, China). The resulting cDNA was subsequently amplified with SYBR Green PCR Master Mix (TransGen, Beijing, China). qRT-PCR was performed and analyzed using an ABI 6900 system (Applied Biosystems, Foster City, United States). The amplification program included an initial denaturation at 95°C for 30 s, followed by 40 cycles of denaturation at 95°C for 5 s and annealing at 60°C for 30 s. Relative expression levels were calculated using the 2^−ΔΔCT^ method, with *β*-actin serving as the housekeeping gene. The sequences of primers used in this study are provided in Table 1.

**Table 1:** Sequences of primers used in the qRT-PCR analysis.

Gene	Forward primer sequence (5′-3′)	Reverse primer sequence (5′-3′)
*GARS1*	ACCTTTGAAAGAGCCCAAAAC	CACTCATCACAAATGGCGAGG
*ATF4*	TCAGACAACAGCAAGGAGGATG	TGGACTAGGGGCTGAAAGAGA
*PHLDB2*	GGAGAAGCACCACCCCAAAG	TGTTGGTGATTATATCCTCTGAGC
*PSAT1*	TGGCAACACCAAAGGAGACG	TGCCTCCCACGGACCTATG
*ERMP1*	CCATTCAGAGAGCAGGTGACAA	CACGAGACGGGTAGGCAATG
*HSP90AA1*	GAAGGTTCGGGAGGCTTCTGG	CTCCTCGGGCATCTTGGCTG
*CALM1*	TGGACGCTGATGGTAATGGC	TATCGAAGACTCGGAACGCC
*ITGA5*	CTCTGTGGCTGTGGGTGAAT	GTAGGAGGCCATCTGTTCCC
*ANGPTL2*	GTGCGACCAGAGACATGACC	CAATGTTCCCAAACCCTTGCTTA
*KPNB1*	ATCAAGAACCCTGACTGGCG	TTAAGGTGGGCATAGCCTGT
*β-actin*	GATATTGCTGCGCTCGTGGT	CATCCCCCACGTACGAGTC

### Statistical analysis

Unless otherwise specified, all data are expressed as mean ± standard deviation and analyzed using SPSS 26 (IBM, Armonk, NY, United States) and RStudio software (v2023.12.1). Continuous variables were initially tested for normality and variance homogeneity. For data that met these assumptions, independent samples analysis of variance (ANOVA) was conducted for preliminary hypothesis testing, followed by post-hoc analysis using the least significant difference (LSD) method. For data that did not meet these criteria, the Kruskal-Walli’s test was employed, with post-hoc comparisons conducted using the Tukey method. A *p*-value of less than 0.05 was considered statistically significant for all hypothesis tests. In transcriptomics analyses, the similarity of gene expression profiles among different groups was assessed using the permutational multivariate analysis of variance (PERMANOVA) method. Differential expression analysis identified significant DEGs based on the criteria of |Log2 (FoldChange)| > 1 and a *p*-value <0.05. For KEGG and GO enrichment analyses, pathways or GO terms with adjusted *p*-values <0.05 were considered significantly enriched. Data visualization was conducted using OriginPro software (v2024), while schematic diagrams and microscopy images were created and enhanced using Adobe Illustrator and Adobe Photoshop (Adobe, San Jose, CA, United States) unless otherwise noted.

## Results

### Effects of rSPD on MDBK cell viability and IBRV infection

**Figure 1:**
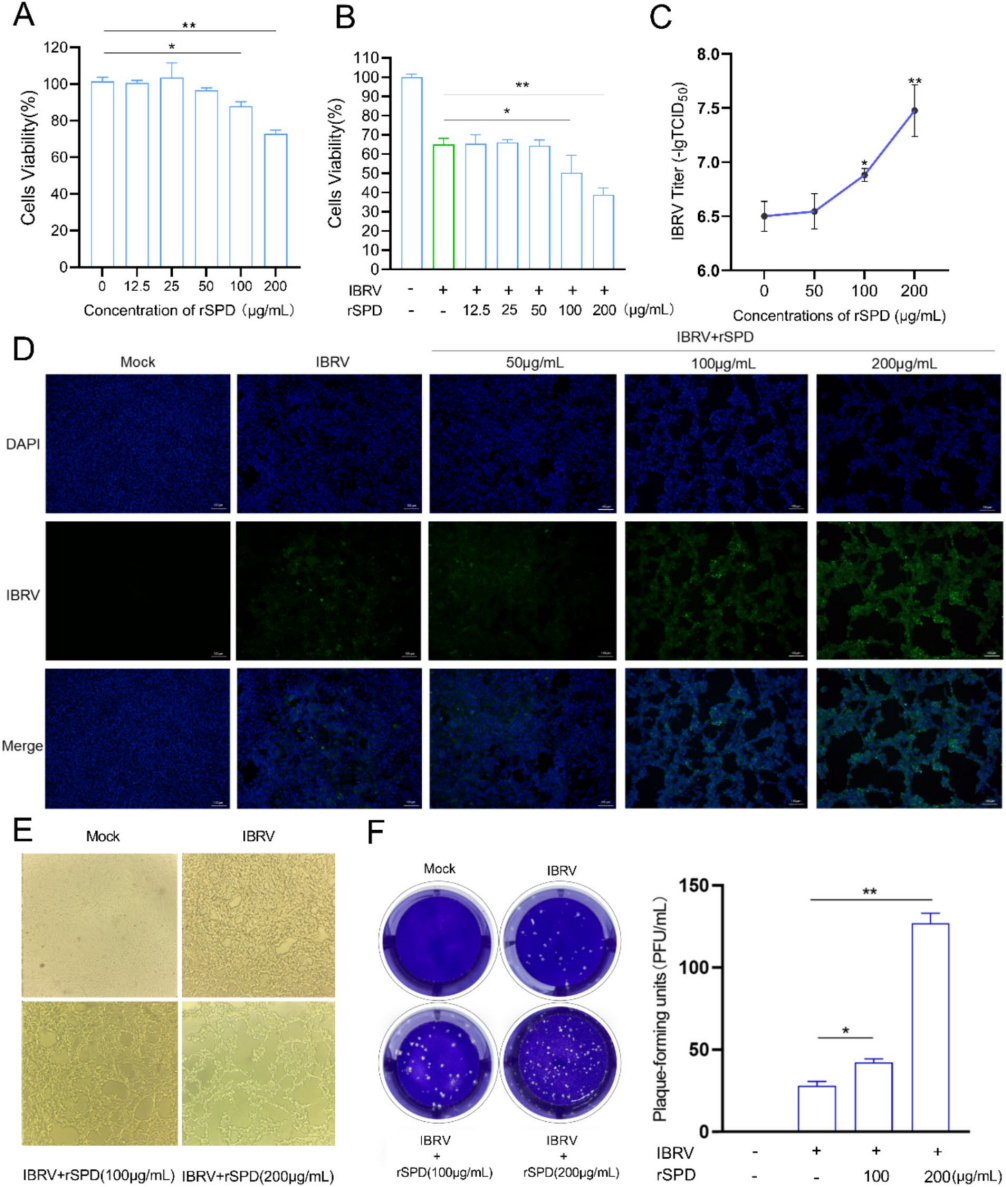
rSPD promotes IBRV proliferation in MDBK cells. **(A,B)** Bar plots showing the viability of MDBK cells treated with various concentrations of rSPD in the absence **(A)** and presence **(B)** of IBRV; **(C)** Bar plot illustrating the viral titers of IBRV in the medium collected from MDBK cells treated with various concentrations of rSPD; **(D)** Representative microscopy images of the stained IBRV in MDBK cells treated with various concentrations of rSPD; **(E)** Representative microscopy images showing the morphology of MDBK cells treated with various concentrations of rSPD after 24 h of IBRV challenge; **(F)** Representative images of plaque formation assay results (left) and a bar plot quantifying plaque-forming units for each treatment (right). In **A–C,F** (right), data are presented as means ± standard deviation. Statistical differences were analyzed using the ANOVA method: **p < 0.01; *p < 0.05.

To evaluate the impact of different concentrations of rSPD on MDBK cell viability, we treated MDBK cells with rSPD at doses of 12.5, 25, 50, 100, and 200 μg/mL for 24 h. The results indicated that exposure to 12.5, 25, and 50 μg/mL rSPD did not significantly affect MDBK cell viability (*p* > 0.05). However, treatment with 100 or 200 μg/mL rSPD significantly reduced MDBK cell viability (*p* < 0.05; Figure 1A).

To further investigate the effect of rSPD on the viability of IBRV-infected MDBK cells, we treated IBRV-infected MDBK cells with the same concentrations of rSPD (12.5, 25, 50, 100, and 200 μg/mL) for 24 h. The results showed that 12.5, 25, and 50 μg/mL rSPD did not significantly alter cell viability compared to the IBRV infection group (*p* > 0.05). In contrast, treatment with 100 μg/mL rSPD significantly reduced cell viability (*p* < 0.05), and treatment with 200 μg/mL rSPD further decreased cell viability (*p* < 0.01) compared to the IBRV infection group (Figure 1B).

To assess the effect of rSPD on IBRV replication, we evaluated viral titers in the medium and MDBK cells. The results demonstrated that treatment with 100 (*p* < 0.05) and 200 (*p* < 0.01) μg/mL rSPD significantly increased the viral titer in the medium, whereas 50 μg/mL rSPD had no significant effect (*p* > 0.05; Figure 1C). Immunofluorescence analysis revealed that, compared to MDBK cells infected with IBRV alone, treatment with 100 and 200 μg/mL rSPD significantly enhanced the fluorescence intensity of IBRV in MDBK cells (*p* < 0.05), while 50 μg/mL rSPD showed no significant effect (Figure 1D).

Optical microscopy observations indicated that control group cells exhibited normal growth, clear contours, good refractive properties, and unchanged morphology and structure. In contrast, MDBK cells infected with IBRV displayed significant pathological changes, including rounding and contraction, vacuole formation, reticular retraction, and grape-like clustering. Specifically, treatment with 100 μg/mL rSPD caused severe cellular damage, including detachment and lysis. Following treatment with 200 μg/mL rSPD, normal cell morphology was completely lost, with extensive lysis and numerous round vacuoles observed (Figure 1E). Additionally, viral plaque assay results showed that treatment 100 and 200 μg/mL rSPD significantly increased (*p* < 0.05) the formation of viral plaques compared to MDBK cells infected with IBRV alone (Figure 1F).

### Effects of rSPD on IBRV replication in MDBK cells

**Figure 2:**
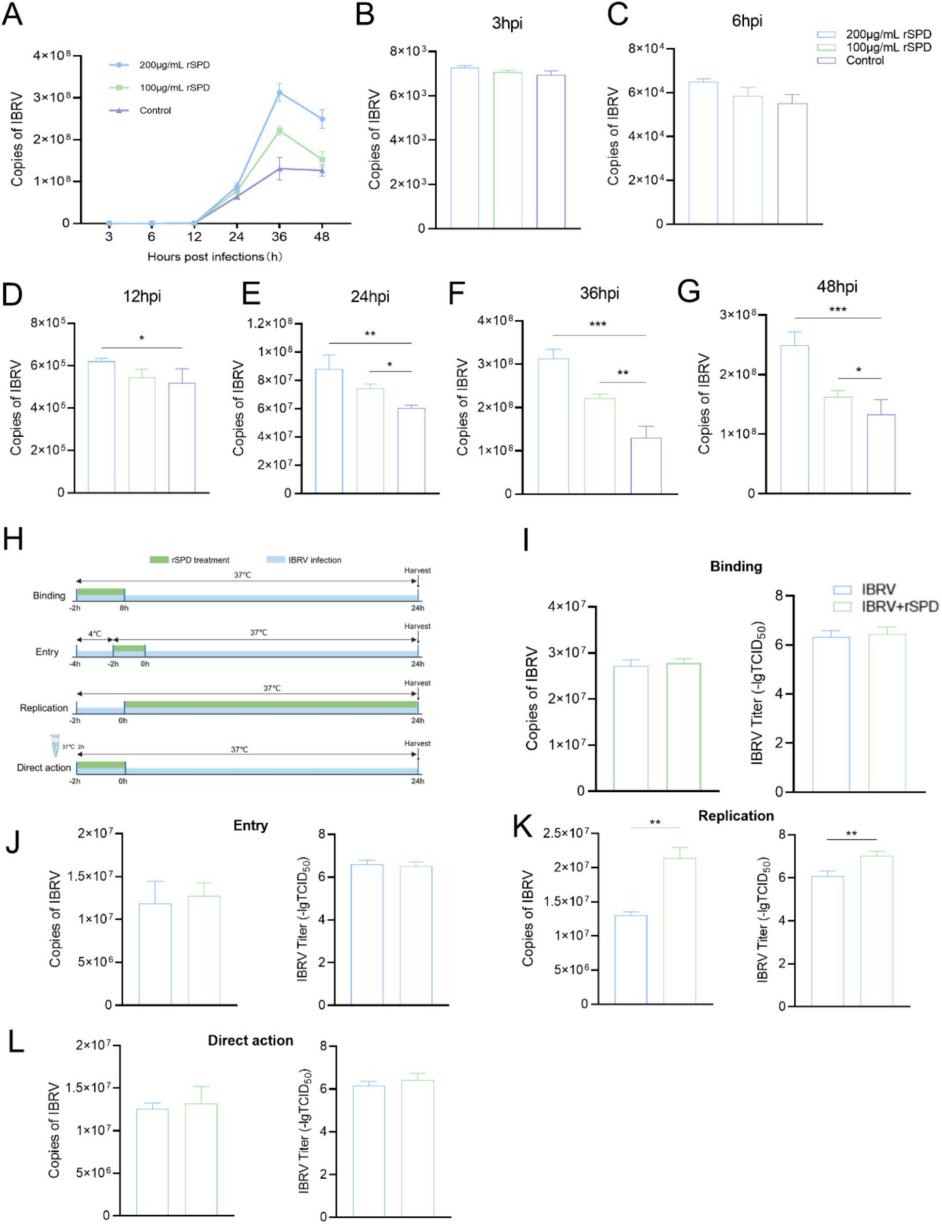
rSPD promotes IBRV replication during the intracellular proliferation phase. **(A)** Dot-line plot showing the copy numbers of the IBRV *gB* gene in the medium collected from MDBK cells at various time points during the 48 h post-infection; B-G: Bar plots illustrating the copy numbers of the IBRV *gB* gene in the medium collected from MDBK cells at 3 **(B)**, 6 **(C)**, 12 **(D)**, 24 **(E)**, 36 **(F)**, and 48 **(G)** hours post-infection; H: Schematic diagram depicting the experimental design used to investigate the effects of rSPD on different stages of IBRV infection; I-K: Bar plots showing the copy numbers of the IBRV *gB* gene and viral titers during the Binding **(I)**, Entry **(J)**, and Replication **(K)** stages; **(L)** Bar plots showing the copy numbers of the IBRV *gB* gene and viral titers resulting from direct interaction with rSPD. In **(A–G,I–L)**, data are presented as means ± standard deviation. Statistical differences were analyzed using the ANOVA method: **p < 0.01; *p < 0.05.

To evaluate the effect of rSPD on the IBRV growth curve, MDBK cells were treated with varying concentrations of rSPD (0, 100, and 200 μg/mL) and challenged with IBRV for time intervals of 3, 6, 12, 24, 36, and 48 h. After incubation, the cells were collected, and the IBRV *gB* gene copy number was quantified using FQ-PCR. As shown in Figure 2A, in the control group, the IBRV *gB* gene copy number progressively increased between 3 and 24 h post-infection (hpi), peaked between 24 and 36 h, and plateaued from 36 to 48 h. In contrast, rSPD treatment significantly increased the IBRV *gB* gene copy number in MDBK cells at 12, 24, 36, and 48 hpi (*p* < 0.05; Figures 2D–G), while no significant changes were observed at 3 and 6 hpi (*p* > 0.05; Figures 2B–C).

To further investigate whether rSPD affects specific stages of the IBRV infection process, including adsorption, entry, and intracellular replication, or exerts a direct effect on viral particles, a detailed experimental protocol was designed (Figure 2H). Specific stages of viral infection were controlled by adjusting the infection temperature, and rSPD was administered at each stage to treat the cells. The results demonstrated that rSPD significantly increased the IBRV *gB* gene copy number and viral titer during the intracellular replication stage (*p* < 0.01; Figure 2K). However, rSPD had no significant effect during the binding stage (*p* > 0.05; Figure 2K) or the entry stage (*p* > 0.05; Figure 2J). Furthermore, rSPD did not alter the IBRV *gB* gene copy number or viral titer through direct interaction with viral particles (*p* > 0.05; Figure 2L).

### Differential expression analysis

To investigate the molecular mechanisms underlying the effects of rSPD on IBRV replication in MDBK cells, we analyzed the gene expression profiles of MDBK cells subjected to various treatments. These treatments included the following groups: CG (MDBK cells cultured for 24 h), SG (MDBK cells treated with 200 μΜ rSPD for 24 h), VG (MDBK cells treated with 0.1 MOI IBRV for 24 h), and SVG (MDBK cells treated with 200 μΜ rSPD and 0.1 MOI IBRV for 24 h).

Transcriptome sequencing yielded 51,415,498, 48,622,667, 48,597,463, and 45,504,010 raw reads in groups CG, SG, VG, and SVG, respectively. After filtering low-quality reads, 47,596,666, 45,209,504, 44,826,961, and 42,222,006 clean reads were obtained, corresponding to 28.56, 27.12, 26.89, and 25.34 G of clean data, respectively. Detailed sequencing data statistics are presented in Supplementary Table 1.

**Figure 3:**
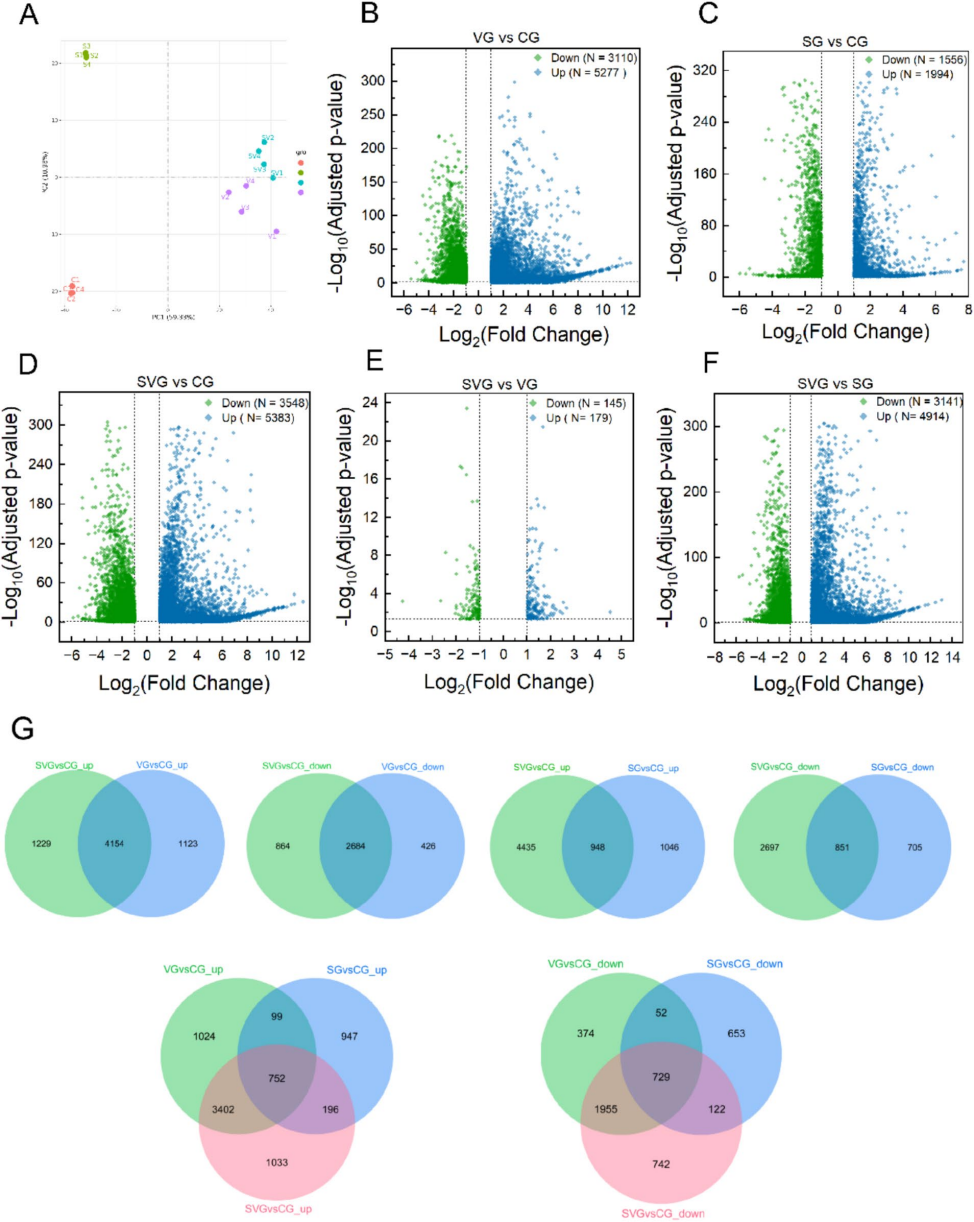
Differential gene expression analysis in MDBK cells across groups. **(A)** Principal component analysis plot illustrating the obtained gene expression profiles of MDBK cells in the group; **(B–F)** Volcano plots displaying differentially expressed genes in comparisons of VG vs. CG **(B)**, SG vs. CG **(C)**, SVG vs. CG **(D)**, SVG vs. VG **(E)**, and SVG vs. SG **(F)**; **(G)** Venn diagrams representing the overlap of differentially expression genes among the treatment groups. In **(B–F)**, blue dots represent significantly up-regulated genes, and green dots represent significantly down-regulated genes (padj < 0.05, |Log2FC| > 1).

In MDBK cells, the expression levels of 5,277, 1994, and 5,383 genes were significantly upregulated compared to the control group following IBRV infection, rSPD treatment, and co-treatment with IBRV and rSPD, respectively. Conversely, the expression levels of 3,110, 1,556, and 3,548 genes were significantly downregulated under the same conditions (Figures 3A–D). When comparing SVG to VG, 179 genes were upregulated and 145 genes were downregulated (Table 2; Figure 3E). Additionally, in the SVG vs. SG comparison, 4,914 upregulated genes and 3,141 downregulated genes were identified (Table 2; Figure 3F). Across all treatments, 4,154 upregulated genes were shared between IBRV-infected cells with and without rSPD treatment, with 1,229 genes uniquely upregulated in IBRV-infected cells and 1,123 genes specifically upregulated in SVG. Similarly, 2,684 downregulated genes were shared, while 864 were unique to VG and 426 were specific to SVG (Figure 3G). Comparison between SVG and SG revealed that 948 upregulated and 851 downregulated genes were shared. Notably, 4,435 upregulated and 2,697 downregulated genes were unique to SVG, while 1,046 upregulated and 705 downregulated genes were exclusive to VG (Figure 3G).

**Table 2:** Number of differentially expressed genes among these groups.

Criteria	Comparisons				
	VG vs CG	SG vs CG	SVG vs CG	SVG vs VG	SVG vs SG
padj < 0.05	Up: 7171	Up: 5439	Up: 7227	Up: 801	Up: 6993
	Down: 5456	Down: 5408	Down: 5603	Down: 825	Down: 5532
	Total: 12627	Total: 10847	Total: 12830	Total: 1626	Total: 12525
padj < 0.05 |Log2FC| > 1	Up: 5277	Up: 1994	Up: 5383	Up: 179	Up: 4914
	Down: 3110	Down: 1556	Down: 3548	Down: 145	Down: 3141
	Total: 8387	Total: 3550	Total: 8931	Total: 324	Total: 8055
padj < 0.05 |Log2FC| > 1.5	Up: 4205	Up: 1062	Up: 4353	Up: 47	Up: 3950
	Down: 1716	Down: 712	Down: 2260	Down: 30	Down: 1806
	Total: 5921	Total: 1774	Total: 6613	Total: 77	Total: 5756

### GO enrichment analysis

**Figure 4:**
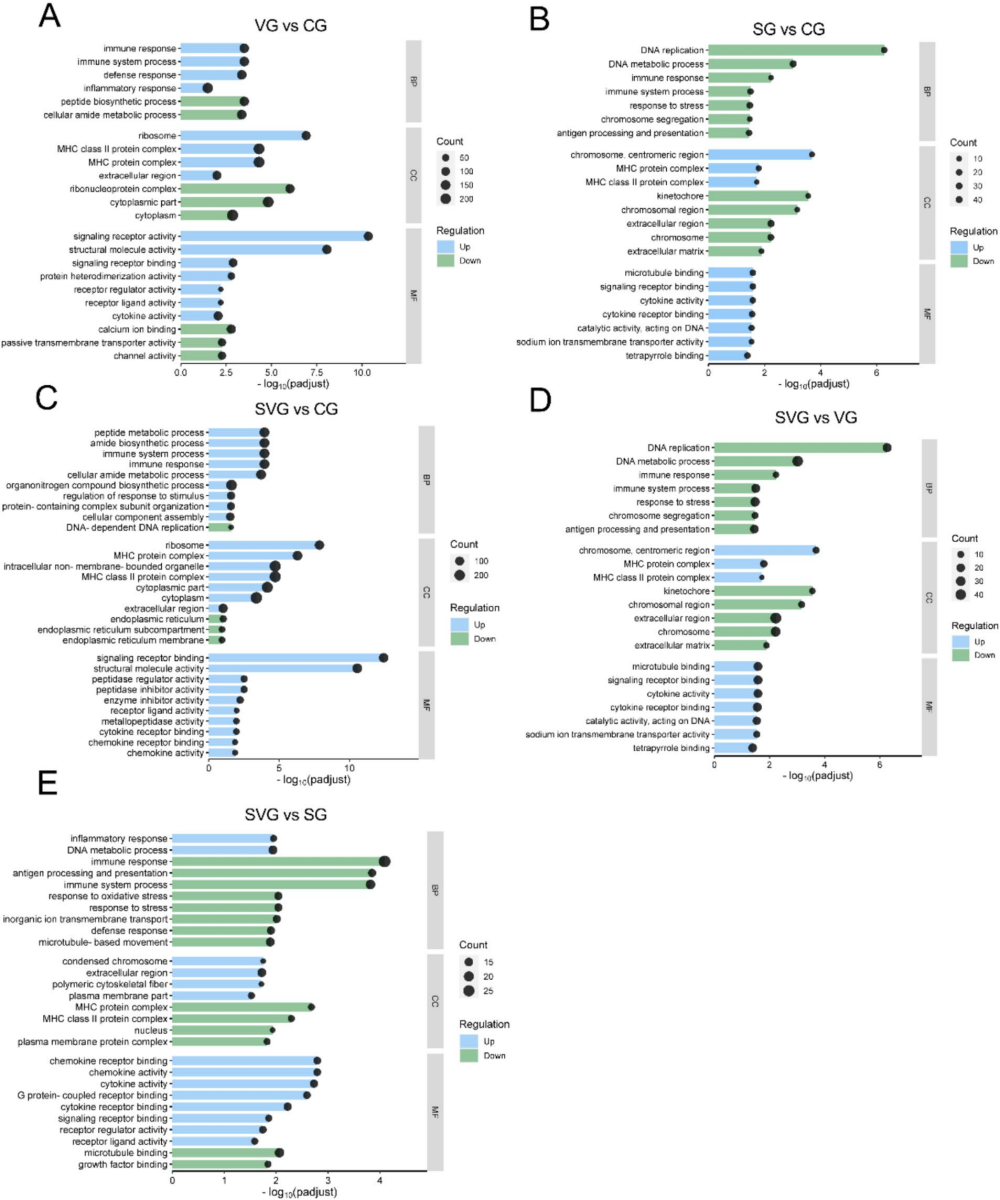
GO enrichment analysis of differentially expressed genes for the comparisons VG vs. CG **(A)**, SG vs. CG **(B)**, SVG vs. CG **(C)**, SVG vs. VG **(D)**, and SVG vs. SG **(E)**. The X-axis represents the negative logarithm of the adjusted *p*-value (padj), while the Y-axis lists the enriched Go terms. The enrichment ratio of genes is shown for GO terms categorized under biological process (BP), cellular components (CC), and molecular function (MF).

The DEGs identified in the VG vs. CG comparison were significantly enriched in GO terms related to immune system processes, immune responses, defense responses, inflammatory responses, cytoplasmic components, MHC protein complexes, signaling receptor activity, structural molecule activity, and signaling receptor binding, among others (Figure 4A). In the SG vs. CG comparison, DEGs were significantly enriched in GO terms associated with host defense responses, such as responses to stress, immune system processes, defense responses, antigen processing and presentation, interspecies interactions, the extracellular region, and the MHC protein complex (Figure 4B). In the SVG vs. CG comparison, DEGs were significantly enriched in GO terms linked to metabolic processes, cellular components, and processes related to cellular immunity and defense responses, such as regulation of immune system processes, immune responses, peptide metabolism, response to stimuli, MHC protein complexes, chemokine activity, and cytokine receptor binding (Figure 4C). DEGs from the SVG vs. VG comparison were significantly enriched in GO terms related to biological processes and cellular components, such as immune responses, antigen processing and presentation, stress responses, inflammatory responses, and MHC protein complexes. Molecular functions such as chemokine activity, cytokine activity, cytokine receptor binding, and signaling receptor binding were also significantly enriched (Figure 4D). Finally, in the SG vs. SVG comparison, DEGs were significantly enriched into GO terms associated with immune responses, antigen processing and presentation, immune system processes, MHC protein complexes, cytokine receptor binding, signaling receptor binding, cytokine activity, and MHC class II protein complexes (Figure 4E).

### KEGG enrichment analysis

**Figure 5:**
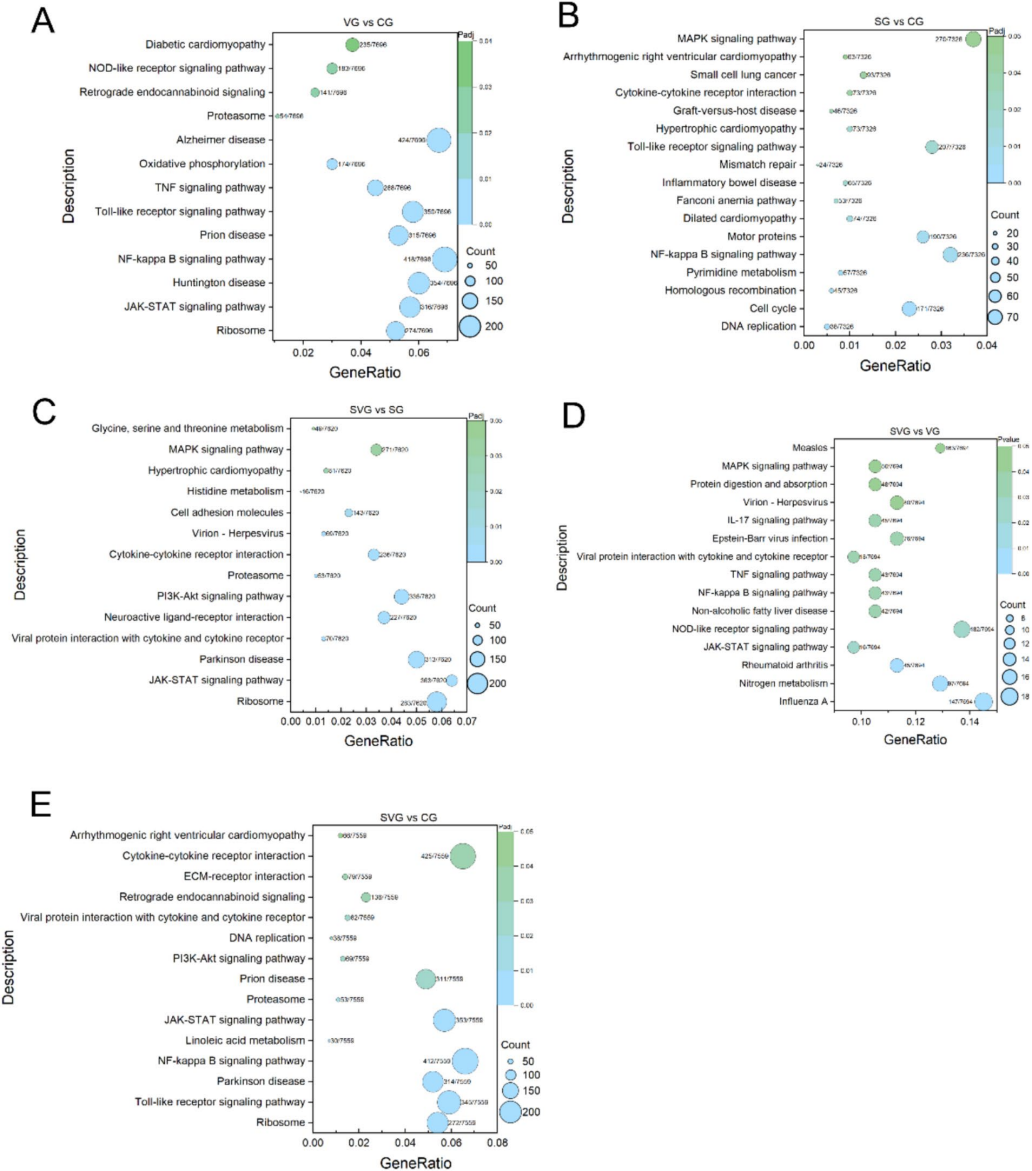
KEGG enrichment analysis of differentially expression genes (DEGs) for the comparisons VG vs. CG **(A)**, SG vs. CG **(B)**, SVG vs. CG **(C)**, SVG vs. VG **(D)**, and SVG vs. SG **(E)**. The Y-axis displays the name of KEGG pathways, while the X-axis indicates the Gene Ratio. “Count” represents the number of DEGs annotated to the corresponding pathway. A higher count indicates that more DEGs enriched are associated with the corresponding pathway. “Gene Ratio” refers to the proportion of DEGs annotated to a specific pathway relative to the total number of DEGs. A larger gene ratio signifies a higher enrichment level of DEGs in that pathway. “Padj” denotes the adjusted *p*-value, which reflects the statistical significance after multiple hypothesis corrections. The color of the symbols corresponds to the adjusted *p*-value, with darker colors indicating greater adjusted *p*-values.

The DEGs identified in the VG vs. CG comparison were significantly enriched in 13 KEGG signaling pathways, including Ribosome, JAK–STAT signaling pathway, Huntington disease, NF-kappa B signaling pathway, Prion disease, Toll-like receptor signaling pathway, TNF signaling pathway, Oxidative phosphorylation, Alzheimer disease, Proteasome, Retrograde endocannabinoid signaling, NOD-like receptor signaling pathway, and Diabetic cardiomyopathy (Figure 5A). In the SG vs. CG comparison, DEGs were significantly enriched in 17 KEGG pathways, including DNA replication, Cell cycle, Homologous recombination, Pyrimidine metabolism, NF-kappa B signaling pathway, Motor proteins, Dilated cardiomyopathy, Fanconi anemia pathway, Inflammatory bowel disease, Mismatch repair, Toll-like receptor signaling pathway, Hypertrophic cardiomyopathy, Graft-versus-host disease, Cytokine-cytokine receptor interaction, Small cell lung cancer, Arrhythmogenic right ventricular cardiomyopathy, and MAPK signaling pathway (Figure 5B). In the SVG vs. CG comparison, DEGs were significantly enriched in 15 KEGG pathways, including Ribosome, Toll-like receptor signaling pathway, Parkinson disease, NF-kappa B signaling pathway, Linoleic acid metabolism, JAK–STAT signaling pathway, Proteasome, Prion disease, PI3K-Akt signaling pathway, DNA replication, Viral protein interaction with cytokine and cytokine receptor, Retrograde endocannabinoid signaling, ECM-receptor interaction, Cytokine-cytokine receptor interaction, and Arrhythmogenic right ventricular cardiomyopathy (Figure 5C). In the SVG vs. VG comparison, DEGs were significantly enriched in 15 KEGG pathways, including Influenza A, Nitrogen metabolism, Rheumatoid arthritis, JAK–STAT signaling pathway, NOD-like receptor signaling pathway, Non-alcoholic fatty liver disease, NF-kappa B signaling pathway, TNF signaling pathway, Viral protein interaction with cytokine and cytokine receptor, Epstein–Barr virus infection, IL-17 signaling pathway, Virion-Herpesvirus, Protein digestion and absorption, MAPK signaling pathway, and Measles (Figure 5D). Finally, in the SVG vs. SG comparison, DEGs were significantly enriched in 14 KEGG pathways, including Ribosome, JAK–STAT signaling pathway, Parkinson disease, Viral protein interaction with cytokine and cytokine receptor, Neuroactive ligand-receptor interaction, PI3K-Akt signaling pathway, Proteasome, Cytokine-cytokine receptor interaction, Virion-Herpesvirus, Cell adhesion molecules, Histidine metabolism, Hypertrophic cardiomyopathy, MAPK signaling pathway, and Glycine, serine, and threonine metabolism (Figure 5E).

### Protein–protein interaction network analysis of DEGs

**Figure 6:**
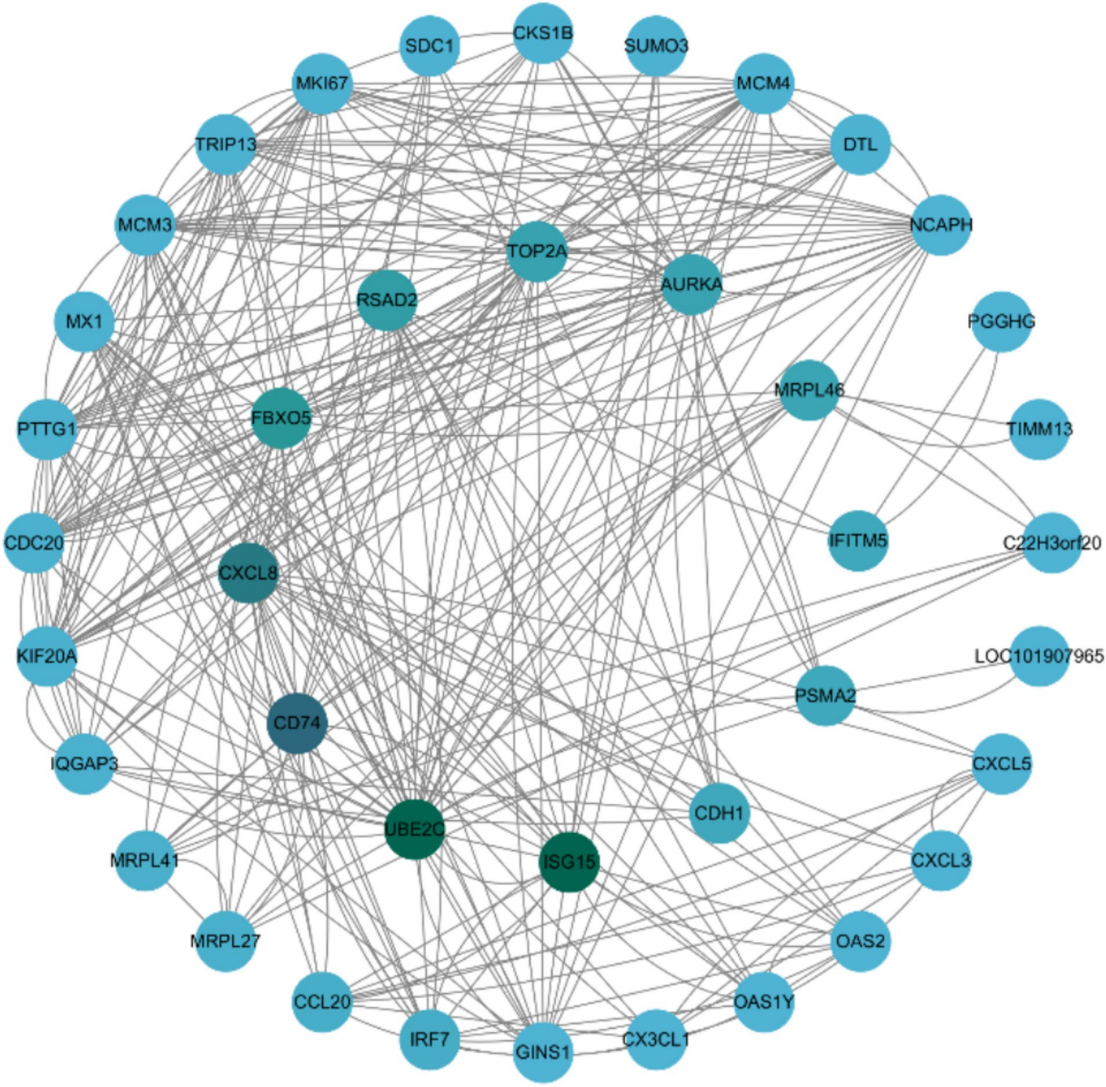
The protein–protein interaction network of differentially expressed genes (DEGs) identified in the SVG vs. VG comparison. The PPI network was constructed using data from the STRING database (http://string-db.org/), which provides interaction relationships for proteins corresponding to the selected DEGs. Only interaction data available in the database for the selected DEGs were extracted and used to construct the network. The network was visualized with Cytoscape (http://cytoscape.org/). The size of the nodes is proportional to the node degree. Nodes are color-coded, with green representing core genes and blue representing peripheral genes.

To further explore the biological significance of the DEGs, a PPI network analysis was performed using the STRING database and visualized with Cytoscape software. The analysis focused on DEGs that met the criteria of FDR ≤ 0.05 and |Log2 Fold Change| ≥ 1 in the SVG vs. VG comparison. A total of 169 interactions involving 49 DEGs were identified within this comparison (Figure 6). Key genes, including *ISG15*, *UBE2C*, *CD74*, *CXCL8*, *FBXO5*, and *RSAD2*, were identified as essential for maintaining the integrity of the network. Furthermore, *ISG15*, *UBE2C*, *CD74*, *CXCL8*, *FBXO5*, *RSAD2*, *IFITM5*, and *IRF7* were highlighted as pivotal contributors to critical biological processes, such as DNA replication, cell cycle regulation, interactions between viral proteins and cytokines or cytokine receptors, cytokine activity, and antiviral defense mechanisms.

### Verification of DEGs by qRT-PCR

**Figure 7:**
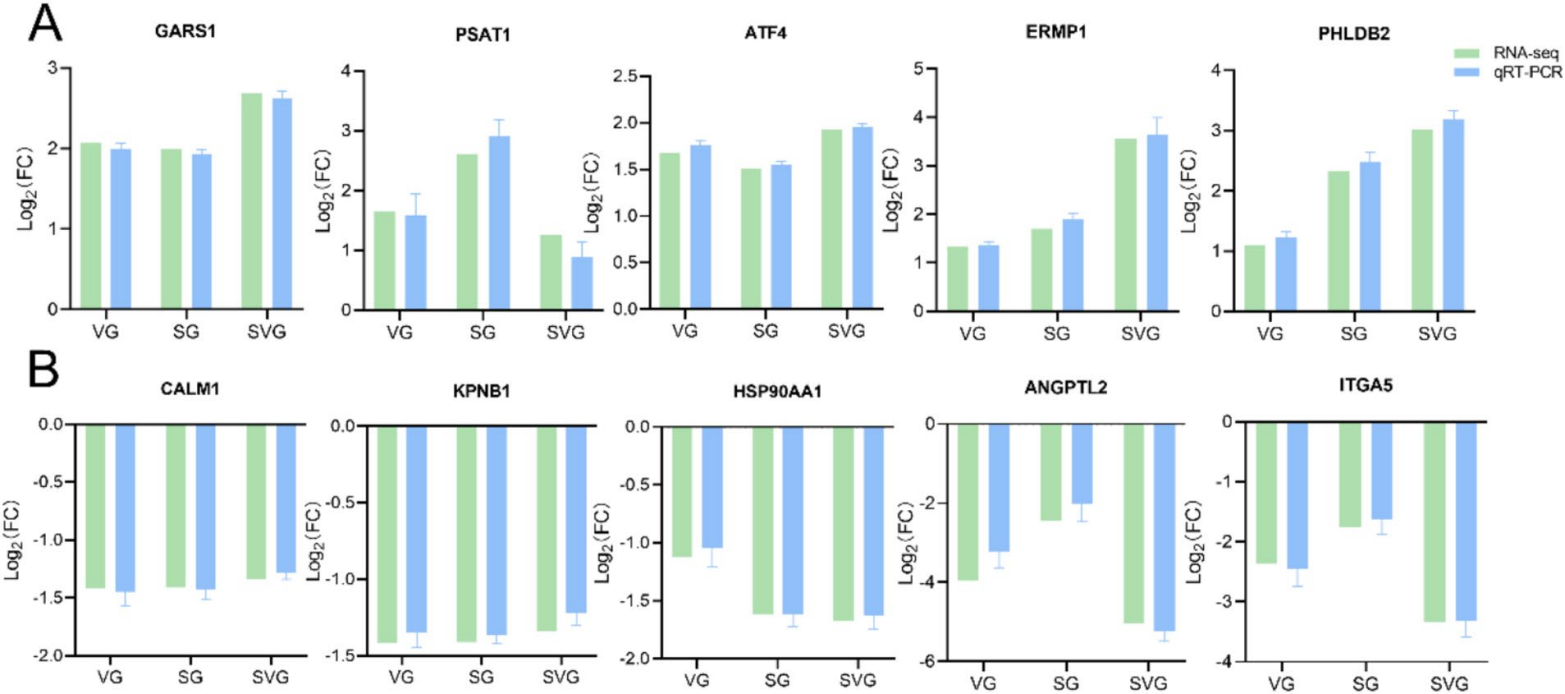
Bar plots showing the Log_2_ fold changes values of 10 randomly selected differentially expressed genes (DEGs) identified as upregulated **(A)** and downregulated **(B)** in the comparison. The expression levels of these DEGs were validated using qRT-PCR, with the *β-actin* gene serving as the internal control. Relative gene expression levels were calculated using the comparative 2^-ΔΔCT^ method (fold change), and the data are expressed as mean ± standard deviations.

To assess the reliability and consistency of the DEGs identified through transcriptome sequencing, 10 genes (*GARS1*, *PSAT1*, *ATF4*, *ERMP1*, *PHLDB2*, *CALM1*, *KPNB1*, *HSP90AA1*, *ANGPTL2*, and *ITGA5*) were randomly selected for qRT-PCR analysis. The results demonstrated that the expression patterns of these genes, either upregulated or downregulated, were consistent with the RNA-seq data, thereby confirming the reliability of the DEGs identified through transcriptome sequencing (Figures 7A,B).

**Figure 8:**
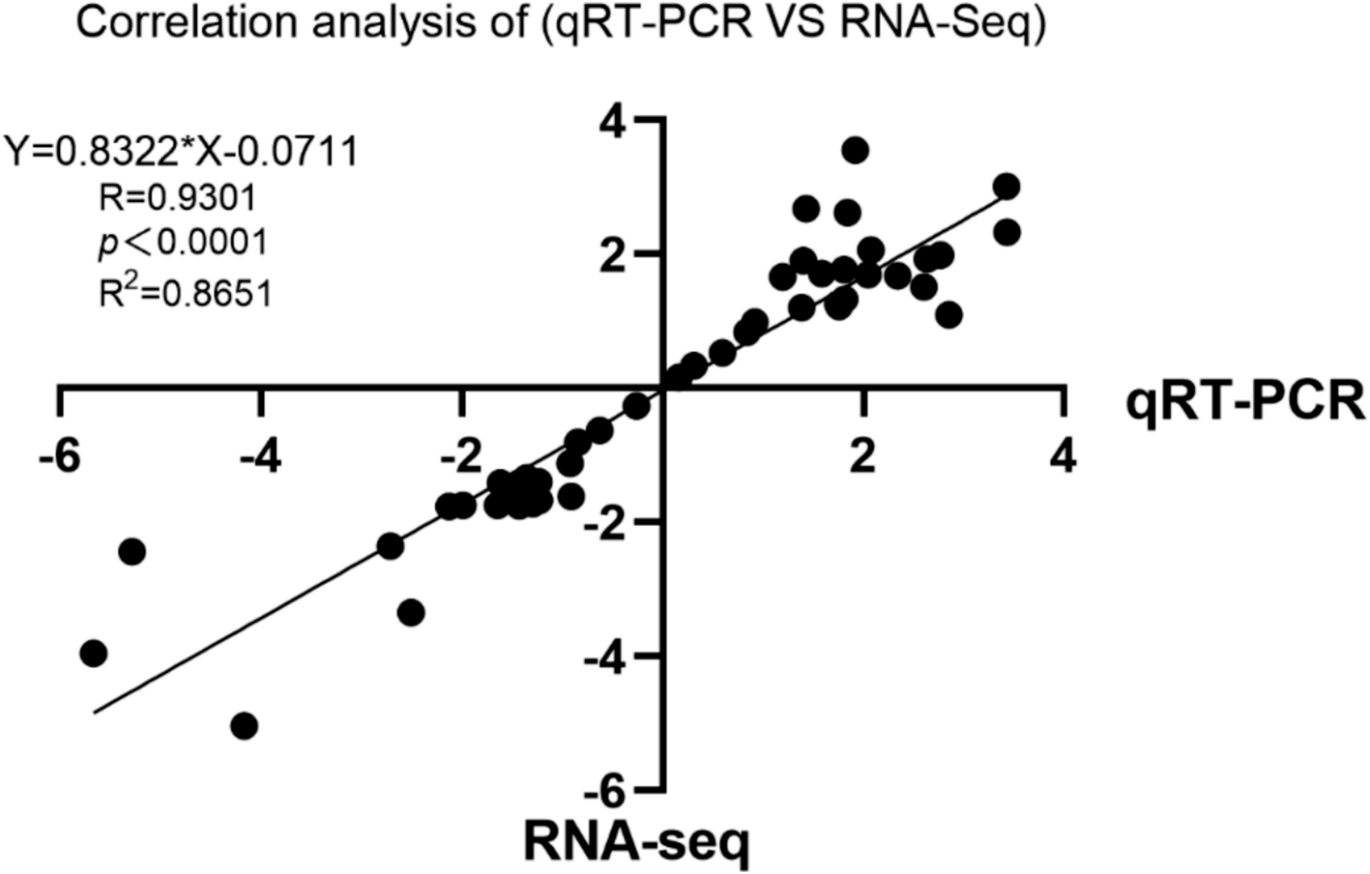
Scatter plot showing the correlation of the fold change values of the 10 randomly selected differentially expressed genes (DEGs).

A correlation analysis was conducted to compare the Log2 fold changes between RNA-seq and qRT-PCR data. The results revealed a strong correlation between the RNA-seq and qRT-PCR data, with an *R*^2^ value of 0.8651 and *p* < 0.0001 (Figure 8).

## Discussion

Bacterial-viral co-infections leading to respiratory diseases have been increasingly severe in recent years, posing a significant threat to both human and animal health (Umar, 2017; Huang et al., 2025; Yadav and Pandey, 2022). IBRV infection is a significant cause of severe respiratory disease in cattle, leading to substantial economic losses in the global cattle industry (Muylkens et al., 2007). Evidence suggests that bacteria can influence viral invasion through various mechanisms, either promoting or suppressing viral infection (Wilks et al., 2013). *S. marcescens* secretes hydrolytic enzymes, such as proteases, lipases, and chitinases. Proteases, as key virulence factors, not only participate in bacterial physiological processes but also affect viral replication and infection (Khaitlina et al., 2020; Lyerly and Kreger, 1983). Currently, there is a limited body of research on co-infection involving IBRV and *Serratia marcescens*; most existing studies focus on *S. marcescens* in conjunction with other viruses. *Serratia marcescens* has been shown to enhance the susceptibility of *Aedes aegypti* mosquitoes to dengue virus infection (Apte-Deshpande et al., 2012). *Serratia marcescens* protease exacerbated influenza virus replication and lung pathology in a mouse model (Akaike et al., 1989). In addition, they can specifically cleave host innate immune effector molecules (such as surfactant protein D), disrupt tight junctions in epithelial cells, and promote bacterial invasion of mammalian cells (Dubin et al., 2013; Potempa and Pike, 2009; Hammers et al., 2022). Based on this background, we first hypothesize that *Serratia marcescens* protease may affect the *in vitro* replication of IBRV, thereby contributing to respiratory disease. In this study, we investigated the effect of rSPD on IBRV replication in MDBK cells and used transcriptomics to explore the underlying molecular mechanisms.

### rSPD enhances the cytotoxicity and replication of IBRV

Serralysin-like proteases, members of the RTX toxin family, are known for their cytotoxic properties and ability to modulate host immune responses (Garcia et al., 2022). In this study, High doses of rSPD significantly reduced the viability of MDBK cells (Figure 1A). Similarly, Molla et al. (1986) demonstrated that the 56 K protease secreted by *Serratia marcescens* exhibited dose-dependent cytotoxicity in cultured human lung fibroblasts. Other studies have shown that serralysin and serralysin-like proteases, such as slpB and slpE, are cytotoxic to HeLa cells, A549 airway cells, and HCLE corneal cells (Marty et al., 2002; Shanks et al., 2015).

It was observed that rSPD further reduced the viability of IBRV-infected cells and enhanced IBRV proliferation in MDBK cells (Figure 1B). Specifically, rSPD treatment increased IBRV titers, promoted viral plaque formation, and intensified immunofluorescence signals in MDBK cells (Figures 1D,E). These findings align with previous studies showing that exposure to bacteria or bacterial components can lead to a 500% increase in poliovirus viral titers and enhanced adhesion of poliovirus to HeLa cells (Kuss et al., 2011). In our study, the copy number of the IBRV *gB* gene progressively increased from the onset of IBRV infection, with rSPD treatment further amplifying this increase (Figure 2A). Similarly, Akaike et al. (1989) reported that protease from *Serratia marcescens* exacerbated influenza virus replication and lung pathology in a mouse model, aligning with our observation. Other studies have also demonstrated that extracellular proteases produced by *Staphylococcus aureus*, *Streptococcus pneumoniae*, and *Haemophilus influenzae* also significantly enhanced viral replication by activating viral proteins or endogenous host proteases (Scheiblauer et al., 1992; Tashiro et al., 1987). The results indicate that rSPD reduces the viability of MDBK cells while promoting the *in vitro* proliferation of IBRV. Interestingly, higher doses of rSPD resulted in greater cytotoxicity but still enhanced IBRV replication. This phenomenon may cause confusion: rSPD promotes IBDV proliferation, or rSPD causes extensive cell death leading to viral proliferation. As shown in Supplementary Figure 2, varying concentrations of rSPD did not affect the viability of MDBK cells during the first 12 h of exposure. However, after 12 h of treatment, a significant increase in the copy number of the IBRV gB gene was observed (Figure 2D). These results indicate that rSPD can promote IBRV replication without immediately compromising cell viability. Another explanation is that while higher doses of rSPD induce cytotoxicity, they may also create a cellular environment that is more permissive to viral replication in surviving cells. Increased cell death and tissue damage could release cellular contents and facilitate viral entry into neighboring, uninfected cells, thus enhancing the spread of the virus (Sattentau, 2008; Britt, 2007). Additionally, rSPD-induced inflammatory responses, such as the release of cytokines or chemokines, might create a favorable niche for viral replication in surviving cells, even if the overall host cell viability is compromised (Mcfarland and Johnson, 1975).

The life cycle of a virus varies significantly depending on its type and classification and generally includes the stages of adsorption, entry, replication, assembly, and release (Jones et al., 2021). Viral infection involves a complex replication cycle, and investigating external factors that affect this cycle is crucial for elucidating the mechanisms that enhance viral infection. In this study, after mixing rSPD with IBRV and incubating at 37°C for 2 h, it was observed that rSPD did not alter the copy number of IBRV in MDBK cells, indicating that rSPD does not directly affect IBRV viral particles (Figure 2L). Similarly, during the virus binding and internalization phases, rSPD did not alter the copy number of IBRV in the cells, suggesting that rSPD does not impact these initial phases of IBRV infection (Figures 2I,J). However, during the intracellular proliferation stage, rSPD was found to significantly increase the viral copy number of the IBRV *gB* gene (Figure 2K), demonstrating that rSPD promotes IBRV replication during this phase. Similar results were also observed in previous studies. For example, Matsuyama et al. (2005) demonstrated that a protease from *Thermus thermophilus*, which facilitates the binding of SARS coronaviruses to the cell surface, increased infection efficiency by 100–1,000 fold. Pyrc et al. (2011) also found that an enzyme from *Porphyromonas gingivalis* enhanced human metapneumovirus infection *in vitro* through direct interaction with viral particles. However, unlike these findings, our results suggest that rSPD promotes IBRV proliferation specifically during the intracellular replication phase, rather than through direct interaction with viral particles or during the binding and internalization stages. We suspect that proteases produced by different bacterial species may exhibit variations in their biological functions due to structural differences (Rao et al., 1998; López-Otín and Bond, 2008), which could account for the distinct mechanisms. On the other hand, as an extracellular protein, rSPD may enter host cells through various mechanisms to exert its effects. During the virus binding stage, rSPD did not significantly affect viral titers or gene copy numbers, potentially because it had not yet entered the cells. The cellular entry mechanism of rSPD remains incompletely understood, but it likely involves receptor-mediated endocytosis, direct membrane translocation, or other forms of receptor-mediated uptake (Brown and Greene, 1991). Previous studies (Rossetto et al., 2000; Geny and Popoff, 2006) have shown that proteins similar to rSPD can enter cells via clathrin-or caveolin-mediated endocytosis; however, the specific pathway utilized by rSPD requires further investigation. In future experiments, cells will be pretreated with rSPD for 24 h prior to viral inoculation to assess whether rSPD influences viral entry or uncoating.

### rSPD induced excessive inflammatory responses in the presence of IBRV

During viral or bacterial infections, pattern recognition receptors (PRRs) detect pathogen-associated molecular patterns (PAMPs) from viruses or bacteria, triggering signaling pathways that induce inflammatory responses. These responses culminate in the production of interferons (IFNs), chemokines, and pro-inflammatory cytokines (Trinchieri, 2010; Macmicking, 2012; Ivashkiv and Donlin, 2014). In this study, numerous immune-related GO terms, including immune response, innate immune response, regulation of apoptotic processes, inflammatory response, and cytokine-mediated signaling pathways, were significantly enriched in the MDBK cells challenged by rSPD and/or IBRV (Figure 4). Similarly, KEGG pathway enrichment analysis revealed that MDBK cells challenged by rSPD and/or IBRV exhibited significantly stronger activation of the MAPK signaling pathway, Toll-like receptor signaling pathway, and NF-κB signaling pathway were significantly enriched by the DEGs identified in the VG vs. CG comparison (Figure 5). Transcriptomic analysis revealed that several pro-inflammatory genes (e.g., *IL-6*, *IL-8*, and *CCL2*) were already upregulated in the VG group, indicating that IBRV infection alone elicits a robust inflammatory response. Upon rSPD treatment (SVG group), these cytokines showed a further increase in expression, suggesting that rSPD amplifies the pre-existing inflammatory response rather than independently initiating it. These findings indicate that rSPD does not function as an autonomous immune modulator but instead potentiates the inflammation induced by IBRV infection. To further investigate this modulatory role, we propose targeted knockdown or pharmacological inhibition of key inflammatory pathways (e.g., NF-κB, JAK–STAT) to assess whether blocking these signaling cascades mitigates the rSPD-mediated enhancement of IBRV replication.

Kida et al. (2007) demonstrated that serralysin from *Serratia marcescens* activates pro-inflammatory signaling pathways via interaction with PAR2 in HeLa cells. Consistent with this, our study showed significantly higher expression levels of *IL-6*, *TLR4*, *IL1β*, *CXCL8*, *S100A8*, *IL-17*, and *CCL2*, in MDBK cells challenged with rSPD, IBRV, and rSPD + IBRV (Supplementary Figures 1A,B; Figure 5). These genes play key roles in pro-inflammatory responses (Wolf et al., 2014; Wu et al., 2015; Boomker et al., 2005; Silva et al., 2021; Wang et al., 2018; Vogl et al., 2007). These results indicate that rSPD exerts a pronounced immunostimulatory or immunoregulatory effect on MDBK cells. However, prolonged activation of PRR pathways can lead to excessive inflammation, resulting in pathological consequences for the host. These include impaired immune function, exacerbation of viral infection-induced tissue damage (Larsen et al., 2020), reduced viral clearance efficiency, and irreversible organ damage, ultimately increasing disease severity and mortality rates (Kumar and Chhibber, 2011; Yang et al., 2023). For example, studies have demonstrated that EBV M81 upregulates CXCL8 expression in target cells, inducing chronic inflammation and subsequently increasing viral production (Li et al., 2019). Similarly, infection with the foot-and-mouth disease virus suppresses LGP2 protein expression, amplifies the inflammatory response, and promotes viral replication (Zhu et al., 2017). Therefore, we propose that the combined effect of rSPD and IBRV induces an exaggerated inflammatory response in MDBK cells, potentially creating a cellular environment conducive to viral replication. However, it is important to note that although rSPD-induced pro-inflammatory signaling may modulate the immune response, this does not necessarily imply a direct enhancement of viral replication. Additional experimental evidence—such as *in vitro* cytokine stimulation assays and detailed mechanistic studies will be required to elucidate the precise relationship between inflammation and increased IBRV replication in this context.

### rSPD impairs antiviral immunity of MDBK cells

Natural immunity serves as the body’s first line of defense against viral infection. Bacteria or their products can influence the course of viral infection or alter the host cell’s immune response, thereby increasing susceptibility to the virus (Karst, 2016). Transcriptome analysis in this study revealed that IBRV infection activated the host cell’s antiviral immune response. This activation was marked by a significant upregulation of interferon-stimulated genes and antiviral-associated proteins, including *ISG15*, *OAS2*, *IFIT1*, *IFIT2*, *IFIT3*, *MX1*, *RSAD2*, *MX2*, *SAA3*, *DDX58*, *IFI44* and *IRF1* (Supplementary Figure 1C). KEGG pathway enrichment analysis further demonstrated that the DEGs in the VG group were enriched in critical pathways such as the JAK–STAT signaling pathway, NF-kappa B signaling pathway, MAPK signaling pathway, and cytokine-cytokine receptor interaction (Figure 5). These findings are consistent with previous studies indicating that IBRV infection upregulates genes involved in innate immune and pro-inflammatory responses (Righi et al., 2023; Levings and Roth, 2013). Type I interferon plays a pivotal role in activating the JAK–STAT signaling pathway, promoting the expression of key cytokines, including signal transducers and activators of transcription (*STAT1*, *STAT2*, *STAT3*) and interferon *α* and β receptor subunits (*IFNAR1* and *IFNAR2*). Among these, STAT1 is a crucial component of the JAK–STAT signaling pathway, essential for initiating the host’s antiviral immune response. However, in this experiment, the expression level of *STAT1* was significantly downregulated following rSPD treatment (Supplementary Figure 1C). This down-regulation suggests that the transcript levels of type I interferon, which are upstream regulators in the JAK–STAT pathway, may be reduced in rSPD-treated infected cells. Despite this, our results indicate that rSPD treatment did not affect the expression levels of type I interferons (*IFNL2* and *IFNL3*) (Supplementary Figure 1C). Interferons exert their antiviral effects by inducing the expression of a range of antiviral effectors through the JAK–STAT signaling pathway, including *MX1*, *OAS*, *ISG15*, *viperin*, and *IFITM3* (Raftery and Stevenson, 2017). Notably, rSPD treatment, compared to the virus-infected cells, resulted in reduced expression levels of these antiviral effectors, including *MX1*, *OAS*, *ISG15*, and *viperin* (Supplementary Figure 1C). For instance, in the context of respiratory viral infections, *Staphylococcus aureus* proteases have been shown to enhance influenza virus replication by cleaving host surfactant proteins, thereby compromising innate defense mechanisms (Loeffler et al., 2013). Similarly, *Streptococcus pneumoniae* proteases modulate the immune response during influenza infection by downregulating host antiviral pathways and facilitating viral entry, ultimately promoting viral replication (Short et al., 2012). These findings highlight the critical role of bacterial proteases in modulating host–pathogen interactions, particularly by disrupting the balance between immune activation and suppression. Patton et al. (2016) reported that *Chlamydia trachomatis* secretes a Chlamydial Protease-like Activity Factor (CPAF), a protease which inhibits p65 translocation, thereby reducing the expression of type I interferons and pro-inflammatory cytokines. Similarly, Tonello and Montecucco (2009) demonstrated that anthrax lethal toxin, a zinc metalloproteinase, hydrolyzes MAPKK, inhibiting the phosphorylation of downstream protein components ERK 1/2, JNK, and p38 MAPK. This disruption impairs the 3 major cellular signaling pathways critical for immunomodulation. In contrast, rSPD appears to exert its effects through a more specific and nuanced mechanism. Unlike other bacterial proteases that primarily function by directly cleaving host immune mediators, rSPD seems to interact with immune cells in a more indirect manner. By promoting the production of pro-inflammatory cytokines, rSPD may create an inflammatory microenvironment that facilitates viral replication. Furthermore, rSPD’s role in modulating the JAK–STAT pathway, specifically by downregulating STAT1, suggests that it may impair the early antiviral immune response, making host cells more permissive to viral replication. Thus, rSPD’s enhancement of IBRV replication likely arises from a combination of immune modulation and altered inflammatory signaling. This mechanism contrasts with that of other bacterial proteases, which typically exert more direct effects on viral entry or immune suppression.

**Figure 9:**
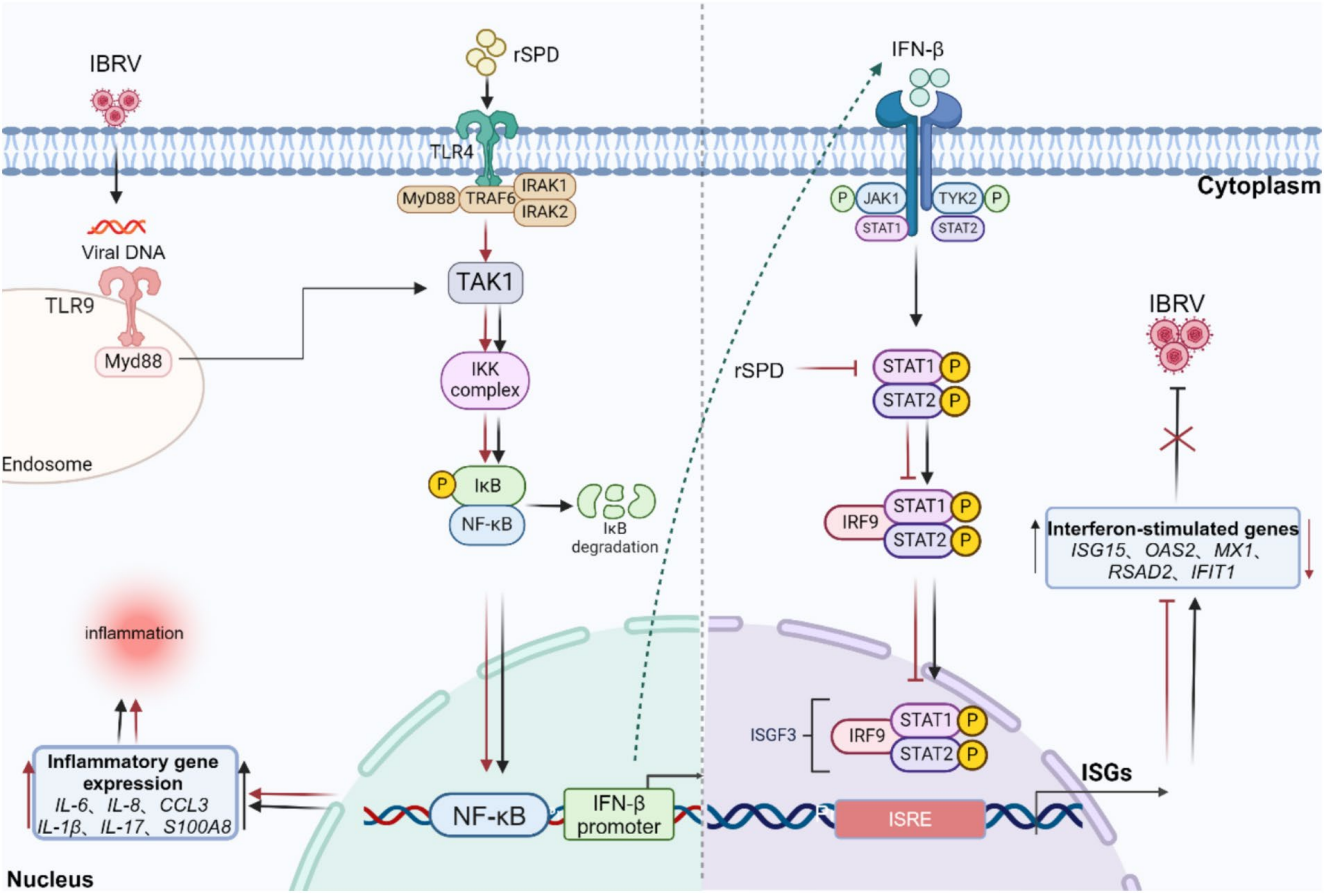
Diagram of the mechanism by which rSPD promotes in vitro replication of IBRV. rSPD and IBRV synergistically activate the NF-kappa B signaling pathway to promote the expression of inflammatory genes (IL-6, IL-8, CCL3, IL-1β, IL-17, S100A8), leading to cellular inflammatory damage. rSPD promotes viral replication by interfering with the JAK–STAT signaling pathway and inhibiting the expression of downstream antiviral effectors (ISG15, OAS2, MX1, RSAD2, IFIT1). The arrows indicate activation, and the blunt-ended lines indicate inhibition. The image was created using the website https://app.biorender.com/.

Additionally, previous research has shown that proteases from *Serratia marcescens* can compromise the host immune response by degrading key humoral immune components, including immunoglobulins (IgA and IgG), α-proteinase inhibitors, and antimicrobial peptides (Molla et al., 1986; Molla et al., 1988). Ishii et al. (2014) demonstrated that proteases from *Serratia marcescens* suppress cellular immunity by impairing the adhesion properties of immune surveillance cells and reducing the phagocytic activity of hemocytes in the hemolymph of the silkworm, *Bombyx mori*. The dual suppression of humoral and cellular immune responses underscores the significant role of *Serratia marcescens* proteases in evading host defenses. These findings suggest that rSPD may affect the host cell’s innate immunity against viral infection by modulating upstream signaling pathways of antiviral effectors, particularly the JAK–STAT signaling pathway (Figure 9). Furthermore, although our transcriptomic data suggest that rSPD suppresses innate immune responses, this alone does not conclusively demonstrate that rSPD directly facilitates IBRV replication. To further investigate this potential mechanism, immunofluorescence and protein co-localization techniques could be employed to assess whether rSPD physically interacts with viral components within the cell. In addition, siRNA or CRISPR-Cas9 will be used to knock out STAT1, JAK1/2, or NF-κB in MDBK cells to evaluate whether inhibition of these pathways directly influences IBRV replication in the presence of rSPD.

Finally, while our study offers important insights into the role of rSPD in promoting IBRV replication *in vitro*, we acknowledge the limitations of extrapolating these findings to *in vivo* contexts. While MDBK cells are a well-established model for IBRV research, they are renal-derived and may not fully replicate the innate immune responses of bovine respiratory epithelial cells, which are the natural target of IBRV in cattle. The use of MDBK cells does not fully capture the complexity of the host response in a whole organism, particularly in the context of bacterial-viral co-infection. The *in vitro* model lacks many of the physiological factors present *in vivo*, such as the immune cell diversity, tissue architecture, and complex cytokine networks that govern host-pathogen interactions. One critical limitation is that *in vitro* studies do not fully replicate the dynamics of viral dissemination, immune activation, or tissue-specific responses that occur during a co-infection. *In vivo* models, such as bovine or small animal systems, would therefore be essential to gain more comprehensive insights into how rSPD influences viral replication within the context of an intact immune system.

## Conclusion

Collectively, our study demonstrates that rSPD promotes IBRV replication during the intracellular proliferation phase by suppressing the host antiviral immune response. Transcriptomic data indicate that rSPD activates inflammatory signaling pathways, which contribute to cellular damage and excessive inflammation. Furthermore, rSPD downregulates the JAK–STAT signaling pathway, inhibiting the expression of key antiviral effectors and thereby facilitating IBRV replication. While increased intracellular viral gene copy numbers were observed, this may also result from altered viral assembly, trafficking, or egress, rather than a direct enhancement of viral replication. These findings provide valuable insights into the complex interplay between bacterial proteases and viral infections. By elucidating how rSPD modulates host immune responses to favor viral replication, this study highlights the potential role of bacterial proteases in exacerbating viral pathogenesis. Future studies, including co-localization of rSPD with viral replication complexes, will be critical in determining the precise molecular mechanisms through which rSPD influences viral replication.

## Data Availability

The datasets presented in this study can be found in online repositories. The names of the repository/repositories and accession number(s) can be found at: https://www.ncbi.nlm.nih.gov/, PRJNA1213708.

## Footnotes

^1^ http://ftp.ensembl.org/pub/release-105/fasta/bos_taurus/

^2^ http://string-db.org/

^3^ http://cytoscape.org/
