# Supplementary material for: Recombinant serralysin metalloproteases D enhances the intracellular replication of infectious bovine rhinotracheitis virus
Source: Front Microbiol. 2025 May 9;16:1567288. doi: 10.3389/fmicb.2025.1567288 (PMC12100627; doi:10.3389/fmicb.2025.1567288)
Supplement: Supplementary file 1 [file Data_Sheet_1.pdf]

Supplementary Table 1 Summary statistics for sequence quality control and mapped data of sample.

Sample	Raw reads	Clean reads	Clean bases	Error rate	Q20(%)	Q30(%)	GC pct	Total mapped	Multiple mapped	Uniquely mapped
CG1	46989090	44048478	6.61G	0.01	98.25	94.96	50.69	42067736	819630	41248106
CG2	63407478	58285906	8.74G	0.01	98.31	95.12	51.56	55177212	1159215	54017997
CG3	46872828	43378994	6.51G	0.01	98.49	95.57	50.42	41073075	876403	40196672
CG4	48392596	44673286	6.7G	0.01	98.4	95.36	51.39	42538294	869296	41668998
SG1	46285332	42749362	6.41G	0.01	98.5	95.61	49.23	40680447	752696	39927751
SG2	47785674	44691346	6.7G	0.01	98.33	95.16	49.76	42456274	818153	41638121
SG3	47942460	44323996	6.65G	0.01	98.29	95.07	49.02	42156750	783762	41372988
SG4	52477202	49073314	7.36G	0.01	98.39	95.31	49.67	46754095	857358	45896737
VG1	49108932	45347372	6.8G	0.02	94.96	88.14	66.88	9480140	257199	9222941
VG2	47854432	44424690	6.66G	0.02	95.34	88.91	64.68	13660204	342415	13317789
VG3	50638474	46672706	7.0G	0.02	95.42	89.18	65.56	12833016	415248	12417768
VG4	46788016	42863076	6.43G	0.02	95.16	88.68	64.37	12853916	384055	12469861
SVG1	43230424	40055338	6.01G	0.02	94.65	87.45	67.29	7855265	260426	7594839
SVG2	46573824	43785410	6.57G	0.02	95.01	88.2	64.99	7420293	231818	7208105
SVG3	42749788	39725870	5.96G	0.02	94.68	87.47	67.55	12065786	212188	11730274
SVG4	49462006	45313648	6.8G	0.02	95.37	89.09	64.99	11790336	293572	11496764

Supplementary Table 2 The results of $TCID_{50}$ testing for IBRV

Dilution ratio(lg)	Repetition	CPE	No CPE	Accumulation of CPE	Accumulation of no CPE	Total	Percentage
-1	8	8	0	48	0	48	100.00%
-2	8	8	0	40	0	40	100.00%
-3	8	8	0	32	0	32	100.00%
-4	8	8	0	24	0	24	100.00%
-5	8	8	0	16	0	16	100.00%
-6	8	6	2	8	2	10	80.00%
-7	8	1	7	2	9	11	18.18%
-8	8	1	7	1	16	17	5.88%
-9	8	0	8	0	24	24	0.00%
-10	8	0	8	0	32	32	0.00%

Supplementary Table 3 Statistics of IBRV-induced MDBK cell lesions by different concentrations of rSPD

Dilution ratios(lg)	Repetition	The concentrations of drugs (μg/mL)			
		0	50	100	200
	1	6	6	6	6
-4	2	6	6	6	6
	3	6	6	6	6
	1	6	6	6	6
-5	2	6	6	6	6
	3	6	6	6	6
	1	5	5	5	6
-6	2	4	4	5	6
	3	5	5	4	6
	1	3	2	3	4
-7	2	1	1	3	3
	3	2	2	2	5
	1	1	0	0	2
-8	2	0	0	0	1
	3	0	0	0	2
	1	0	0	0	0
-9	2	0	0	0	0
	3	0	0	0	0

Supplementary Table 4 KEGG pathway classification of differently expressed genes (VG vs CG)

Description	Pathway ID	DEGs	P-Value
Ribosome	bta03010	170	5.41E-11
JAK-STAT signaling pathway	bta05012	188	8.32E-10
Huntington disease	bta05016	197	4.41E-07
NF-kappa B signaling pathway	bta05014	226	1.25E-06
Prion disease	bta05020	175	2.27E-06
Toll-like receptor signaling pathway	bta05171	190	6.42E-06
TNF signaling pathway	bta04714	147	3.58E-05
Oxidative phosphorylation	bta00190	100	5.82E-05
Alzheimer disease	bta05010	220	6.70E-05
Proteasome	bta03050	35	0.000881
Retrograde endocannabinoid signaling	bta04723	79	0.000971
NOD-like receptor signaling pathway	bta04932	99	0.001211
Diabetic cardiomyopathy	bta05415	123	0.001754
Chemical carcinogenesis - reactive oxygen species	bta05208	134	0.002409
ECM-receptor interaction	bta04512	47	0.002821
DNA replication	bta03030	25	0.003523
Cell cycle	bta04110	91	0.004431
Nicotine addiction	bta05033	17	0.005115
Epstein-Barr virus infection	bta05412	39	0.007716
Legionellosis	bta05134	40	0.010671
Dilated cardiomyopathy	bta05414	47	0.017298

Purine metabolism	bta00230	70	0.02031
Cardiac muscle contraction	bta04260	47	0.022282
Pentose phosphate pathway	bta00030	17	0.027616
Hypertrophic cardiomyopathy	bta05410	44	0.030263
Spinocerebellar ataxia	bta05017	75	0.036967
Cysteine and methionine metabolism	bta00270	32	0.038354
Pyruvate metabolism	bta00620	25	0.042369
Protein digestion and absorption	bta04974	47	0.044263

Supplementary Table 5 KEGG pathway classification of differently expressed genes (SG vs CG)

Description	Pathway ID	DEGs	P-Value
DNA replication	bta04110	59	5.18E-20
Cell cycle	bta03030	26	1.19E-18
Homologous recombination	bta03440	20	7.40E-10
Pyrimidine metabolism	bta03460	21	3.58E-09
NF-kappa B signaling pathway	bta05014	73	1.86E-08
Motor proteins	bta03040	40	2.43E-08
Dilated cardiomyopathy	bta04814	41	2.53E-07
Fanconi anemia pathway	bta03430	12	4.07E-07
Inflammatory bowel disease	bta03410	16	1.06E-06
Mismatch repair	bta04114	29	1.33E-06
Toll-like receptor signaling pathway	bta00240	18	2.44E-06
Hypertrophic cardiomyopathy	bta05020	51	3.99E-05
Graft-versus-host disease	bta04914	21	6.66E-05
Cytokine-cytokine receptor interaction	bta03013	27	7.72E-05
Small cell lung cancer	bta05012	48	0.000279473
Arrhythmogenic right ventricular cardiomyopathy	bta01232	19	0.000879099
MAPK signaling pathway	bta03420	14	0.002075465
Cellular senescence	bta00983	13	0.002766897
Hematopoietic cell lineage	bta00230	22	0.003086457
Nucleotide metabolism	bta05016	48	0.003380084
Gap junction	bta04540	15	0.004205951
Thermogenesis	bta04714	38	0.004621143
Estrogen signaling pathway	bta04915	18	0.005106259

Supplementary Table 6 KEGG pathway classification of differently expressed genes (SVG vs CG)

Description	Pathway ID	DEGs	P-Value
Ribosome	bta03010	193	3.87E-16
Toll-like receptor signaling pathway	bta05171	210	9.40E-08
Parkinson disease	bta05012	186	6.53E-06
NF-kappa B signaling pathway	bta05014	234	2.91E-05
Linoleic acid metabolism	bta00591	25	4.50E-05
JAK-STAT signaling pathway	bta05016	201	8.71E-05
Proteasome	bta03050	38	0.000232031

Prion disease	bta05020	175	0.00054905
PI3K-Akt signaling pathway	bta05134	46	0.000760681
DNA replication	bta03030	28	0.000765647
Viral protein interaction with cytokine and cytokine receptor	bta05414	53	0.000956061
Retrograde endocannabinoid signaling	bta04723	82	0.001199853
ECM-receptor interaction	bta04512	51	0.001233442
Cytokine-cytokine receptor interaction	bta05010	230	0.001592424
Arrhythmogenic right ventricular cardiomyopathy	bta05412	43	0.002238081
Hypertrophic cardiomyopathy	bta05410	51	0.002750522
Transcriptional misregulation in cancer	bta05202	98	0.005263374
Chemical carcinogenesis - reactive oxygen species	bta05208	141	0.007733244
Ras signaling pathway	bta03060	17	0.008260132
Cell cycle	bta04110	96	0.009952364
Galactose metabolism	bta00052	20	0.014191202
Fatty acid elongation	bta00062	20	0.014191202
Protein digestion and absorption	bta04974	53	0.015545516
Nucleocytoplasmic transport	bta03013	74	0.018208683
Ether lipid metabolism	bta00565	27	0.018220745
Oxidative phosphorylation	bta00190	96	0.018241169
Carbon metabolism	bta01200	68	0.026313365
Thermogenesis	bta04714	140	0.027733767
Pentose phosphate pathway	bta00030	18	0.03168309
Vascular smooth muscle contraction	bta04270	60	0.037238284
Arachidonic acid metabolism	bta00590	34	0.037642176
Ras signaling pathway	bta04014	119	0.037708076
Citrate cycle (TCA cycle)	bta00020	20	0.038045163
Propanoate metabolism	bta00640	20	0.038045163
p53 signaling pathway	bta04115	48	0.039914342
Fructose and mannose metabolism	bta00051	22	0.043747953
Biosynthesis of amino acids	bta01230	44	0.047778339

Supplementary Table 7 KEGG pathway classification of differently expressed genes (SVG vs VG)

Description	Pathway ID	DEGs	P-Value
Influenza A	bta04514	18	2.49E-06
Nitrogen metabolism	bta05323	16	4.55E-05
Rheumatoid arthritis	bta05144	14	0.00575008
JAK-STAT signaling pathway	bta03266	12	0.02667823
NOD-like receptor signaling pathway	bta04932	17	0.027411053
Non-alcoholic fatty liver disease	bta05219	13	0.029722008
NF-kappa B signaling pathway	bta04930	13	0.031585034
TNF signaling pathway	bta05332	13	0.031585034
Viral protein interaction with cytokine and cytokine receptor	bta00910	12	0.033312241
Epstein-Barr virus infection	bta04061	14	0.033888722
IL-17 signaling pathway	bta05330	13	0.035490749
Virion - Herpesvirus	bta04612	14	0.039800153
Protein digestion and absorption	bta04940	13	0.041793822
MAPK signaling pathway	bta05320	13	0.046287712

Measles	bta05164	10	0.047703813

Supplementary Table 8 KEGG pathway classification of differently expressed genes (SVG vs SG)

Description	Pathway ID	DEGs	P-Value
Ribosome	bta03010	186	1.91E-16
JAK-STAT signaling pathway	bta05171	106	4.40E-09
Parkinson disease	bta05012	160	0.000526508
Viral protein interaction with cytokine and cytokine receptor	bta04061	42	0.00169819
Neuroactive ligand-receptor interaction	bta04080	117	0.001955063
PI3K-Akt signaling pathway	bta04151	141	0.501175452
Proteasome	bta03050	33	0.002213886
Cytokine-cytokine receptor interaction	bta04060	105	0.002307227
Virion - Herpesvirus	bta05134	40	0.005133773
Cell adhesion molecules	bta04514	75	0.006834622
Histidine metabolism	bta00340	12	0.007691345
Hypertrophic cardiomyopathy	bta05410	45	0.00903396
MAPK signaling pathway	bta04010	108	0.781033986
Glycine, serine and threonine metabolism	bta00260	29	0.011002512
IL-17 signaling pathway	bta05020	151	0.012737757
Amyotrophic lateral sclerosis	bta05014	194	0.013821058
Hematopoietic cell lineage	bta04640	47	0.014959649
Pentose phosphate pathway	bta00030	17	0.022336465
Alzheimer disease	bta05010	199	0.02574504
Apoptosis - multiple species	bta04512	43	0.027613784
Retrograde endocannabinoid signaling	bta04723	69	0.027654623
Phagosome	bta04145	81	0.03125468
Complement and coagulation cascades	bta04610	38	0.031935553
Oxidative phosphorylation	bta00190	86	0.036343696
Regulation of actin cytoskeleton	bta04810	102	0.038701858
Leukocyte transendothelial migration	bta04670	51	0.041357683
Arrhythmogenic right ventricular cardiomyopathy	bta05412	35	0.04479921
Glycolysis / Gluconeogenesis	bta00010	34	0.046194161

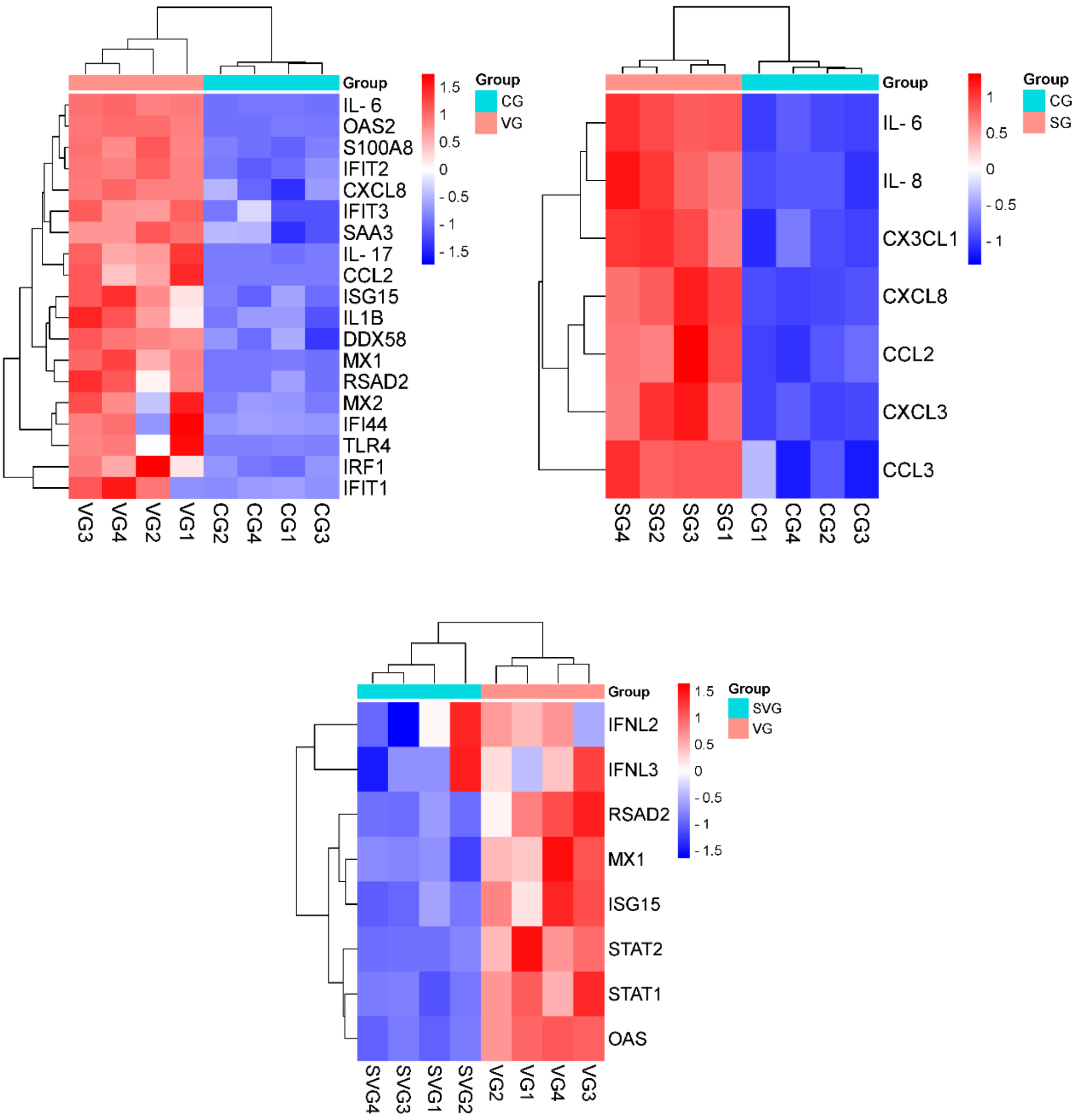

Supplementary Figure 1. (A) Clustering plot of some genes in the VG and CG groups. (B) Clustering plot of selected inflammatory genes in the SG versus CG group. (C) Clustering plot of selected antiviral genes in SVG and VG groups. Red color indicates high expression of a single gene and blue color indicates low expression of a single gene.

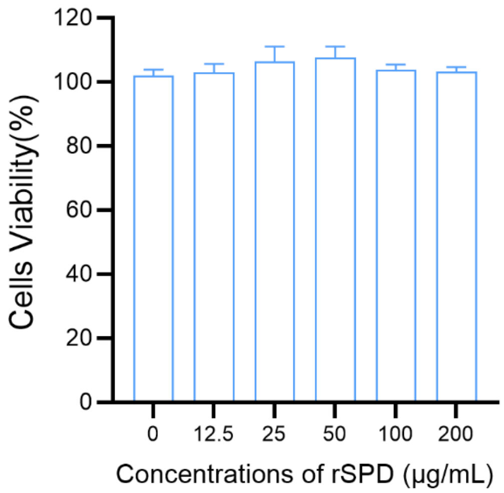

Supplementary Figure 2. Effects of different concentrations of rSPD on the survival rate of MDBK cells after 12 h
